# Determination of superior *Pistacia chinensis* accession with high-quality seed oil and biodiesel production and revelation of LEC1/WRI1-mediated high oil accumulative mechanism for better developing woody biodiesel

**DOI:** 10.1186/s12870-023-04267-y

**Published:** 2023-05-19

**Authors:** Feng Chen, Weijun Lin, Wei Li, Jinhe Hu, Zhi Li, Lingling Shi, Zhixiang Zhang, Yu Xiu, Shanzhi Lin

**Affiliations:** 1 Beijing Advanced Innovation Center for Tree Breeding By Molecular Design, College of Biological Sciences and Biotechnology, School of Soil and Water Conservation, National Engineering Laboratory for Tree Breeding, Key Laboratory of Genetics and Breeding in Forest Trees and Ornamental Plants, Ministry of Education, Tree and Ornamental Plant Breeding and Biotechnology Laboratory of National Forestry and Grassland Administration, Beijing Forestry University, Beijing, 100083 China; 2 Institute of Botany, Chinese Academy of Sciences, Beijing, 100093 China

**Keywords:** Woody biodiesel, Fuel properties, High oil accumulation, *Pistacia chinensis* seed, Provenance selection, LEC1/WRI1-mediated regulation mechanism

## Abstract

**Background:**

Based on our previous studied on different provenances of *Pistacia chinensis*, some accessions with high quality and quantity of seed oils has emerged as novel source of biodiesel. To better develop *P. chinensis* seed oils as woody biodiesel, a concurrent exploration of oil content, FA profile, biodiesel yield, and fuel properties was conducted on the seeds from 5 plus germplasms to determine superior genotype for ideal biodiesel production. Another vital challenge is to unravel mechanism that govern the differences in oil content and FA profile of *P. chinensis* seeds across different accessions. FA biosynthesis and oil accumulation of oil plants are known to be highly controlled by the transcription factors. An integrated analysis of our recent transcriptome data, qRT-PCR detection and functional identification was performed as an attempt to highlight LEC1/WRI1-mediated transcription regulatory mechanism for high-quality oil accumulation in *P. chinensis* seeds.

**Results:**

To select ideal germplasm and unravel high oil accumulative mechanism for developing *P. chinensis* seed oils as biodiesel, five plus trees (accession PC-BJ/PC-AH/PC-SX/PC-HN/PC-HB) with high-yield seeds were selected to assess the variabilities in weight, oil content, FA profile, biodiesel yield and fuel property, revealing a variation in the levels of seed oil (50.76–60.88%), monounsaturated FA (42.80–70.72%) and polyunsaturated FA (18.78–43.35%), and biodiesel yield (84.98–98.15%) across different accessions. PC-HN had a maximum values of seed weight (26.23 mg), oil (60.88%) and biodiesel yield (98.15%), and ideal proportions of C18:1 (69.94%), C18:2 (17.65%) and C18:3 (1.13%), implying that seed oils of accession PC-HN was the most suitable for ideal biodiesel production. To highlight molecular mechanism that govern such differences in oil content and FA profile of different accessions, a combination of our recent transcriptome data, qRT-PCR detection and protein interaction analysis was performed to identify a pivotal role of LEC1/WRI1-mediated transcription regulatory network in high oil accumulation of *P. chinensis* seeds from different accessions. Notably, overexpression of *PcWRI1* or *PcLEC1* from *P. chinensis* seeds in Arabidopsis could facilitate seed development and upregulate several genes relevant for carbon flux allocation (plastidic glycolysis and acetyl-CoA generation), FA synthesis, TAG assembly and oil storage, causing an increase in seed oil content and monounsaturated FA level, destined for biodiesel fuel property improvement. Our findings may present strategies for better developing *P. chinensis* seed oils as biodiesel feedstock and bioengineering its high oil accumulation.

**Conclusions:**

This is the first report on the cross-accessions assessments of *P. chinensis* seed oils to determine ideal accession for high-quality biodiesel production, and an effective combination of *PcWRI1* or *PcLEC1* overexpression, morphological assay, oil accumulation and qRT-PCR detection was applied to unravel a role of LEC1/WRI1-mediated regulatory network for oil accumulation in *P. chinensis* seeds, and to highlight the potential application of *PcWRI1* or *PcLEC1* for increasing oil production. Our finding may provide new strategies for developing biodiesel resource and molecular breeding.

**Supplementary Information:**

The online version contains supplementary material available at 10.1186/s12870-023-04267-y.

## Background

Energy crisis, climate warming, and environment deterioration are still the most severe issues for the world, and these problems should be bound to intensify with the heavy use of fossil energy, population expansion and industrialization acceleration [1–3]. Hence, effective exploitation and full development of novel renewable/clean energy has become an urgent issue for sustainable economic development and global energy security. Biodiesel, known as FA methyl ester (FAME), has gradually attracted global great attention as ecofriendly fuel for its nontoxicity and biodegradability [4], which has been widely applied in several countries such as USA, Brazil, Netherlands, Germany, France and Malaysia [5]. Yet, more than 95% of world biodiesel is produced from edible oils (such as sunflower, peanut and soybean) [1, 6–8], which should inevitably lead to reduction of available arable land and shortage of food or edible oils. Thus, exploiting an alternative biodiesel feedstock of non-food plant resources is utmost vital, especially in China with huge population and low per capita arable land. In recent years, the oils from some woody plants (such as *Prunus sibirica*, *Xanthoceras sorbifolia*, *Pistacia chinensis* and *Jatropha curcas*) have been applied as raw material for biodiesel production in China [9–16]. Notably, the Chinese government has announced to achieve the peak of carbon dioxide emissions by 2030 and carbon neutrality before 2060 at the 75th session of the UN General Assembly (UNGA) in 2020. All these will speed up to develop economical non-food plant resources for bioenergy in China.

*Pistacia chinensis* Bunge, one perennial deciduous tree of the Anacardiaceae family, is widely distributed in wild mountain areas (geographical coordinates approximately E96°52′-123°14′, N18°09′-40°09′) in China. It was estimated that the total area of artificial plantation and natural forest of *P. chinensis* was about 86.6 and 133,300 ha, respectively, and the annual yield of *P. chinensis* seeds was about 330,000 tons in China [11, 17–19]. There are many germplasms of *P. chinensis* with different oil content and FA compositions, but some superior germplasms have not been exploited. Based on our previous studies on different germplasms of *P. chinensis*, some accessions have been identified with rich oil content and high proportion of oleic and linoleic acid [17–19], and notably, the oil content (42.5–50.0%) of mature seeds, which was higher than that of Chinese traditional oil woody plants, such as *J. curcas* (38.1%), *L. glauca* (31.6%) and *Comus wilsonian*a (34.5%) [13, 16, 20]. All these revealed that *P. chinensis* seed oil could be as potential biodiesel feedstock in China. Considering the differences in seed oil content and FA profiles of different germplasms, it is vitally important to select superior accession with high-quality seed oil for better development of woody biodiesel. However, an interesting open challenge is the mechanism that governed such differences in oil content and FA compositions of *P. chinensis* seeds across different accessions. Therefore, exploration of molecular regulation mechanism for high oil production in *P. chinensis* seeds has become another one crucial issue for developing woody biodiesel.

It is known that oil biosynthetic process is composed of carbon source supply, FA synthesis, triacylglycerol (TAG) assembly and oil storage involved in several regulatory enzymes [21], and their expressions have been identified to be highly regulated by a series of transcription factors (TFs) [22–30]. Hence, identification of some critical TFs essential for oil accumulation in *P*. *chinensis* seeds could help to unravel TF-mediated molecular regulatory mechanism of increased oil production and may present new strategy for bioengineering oil accumulation in oil plants. Recently, we performed transcriptome sequencing of *P. chinensis* seeds (SRP030781) to identify some TFs in relation to oil accumulation [17], which could make us possible to gain better insight into transcriptional mechanism for governing differences in seed oil content and FA compositions across different germplasms of *P. chinensis*.

This work was undertaken to determine ideal germplasm and to unravel transcriptional regulatory mechanism of high oil production for better development of *P*. *chinensis* seed oils as potential feedstock for biodiesel, To this achieve, 5 plus trees (germplasm accessions PC-BJ, PC-AH, PC-SX, PC-HN and PC-HB) with high-yield seeds were selected for the cross-accessions evaluations of seed phenotypic characteristics (size and weight), oil accumulation (content, FA profile, and oil body amount), biodiesel yield and fuel property of the seeds from different accessions. Such exploration could help to select ideal accession for developing biodiesel. Another vital focus of this work was to highlight transcriptional mechanism that govern such difference in seed oil content and FA composition across different accessions, and to gain useful information for bioengineering oil accumulation or molecular-assisted selection. To this set, based on our recent transcriptome result of *P*. *chinensis* seeds, the comparative assay of cross-accessions correlation of transcript level of genes for the TFs by qRT-PCR with oil accumulative amount, in combination with the protein interaction assay between the TFs and oil-synthesis enzymes, was described as an initial attempt to identify LEC1/WRI1-mediated network crucial for regulating transcriptions of enzymes relevant for FA synthesis and oil accumulation in *P*. *chinensis* seeds of different accessions. Finally, a concurrent exploration was made on dynamic patterns of seedling growth, seed development, oil accumulation (content and FA profile) and some vital gene transcript relevant to FA biosynthesis, TAG assembly and oil storage in transgenic Arabidopsis lines with ectopic overexpression of *WRI1* or *LEC1* from *P. chinensis* seeds, with the aim of gaining a better insight into LEC1/WRI1-mediated transcriptional regulation mechanism for high oil production in *P*. *chinensis* seeds. Our findings could facilitate the development of* P*. *chinensis* seed oils as biodiesel feedstock, and provide new molecular target (WRI1 or LEC1) for bioengineering oil accumulation in oilseed plants.

## Result

### Variability in seed size and weight of 5 selected accessions of* P. chinensis*

**Fig. 1:**
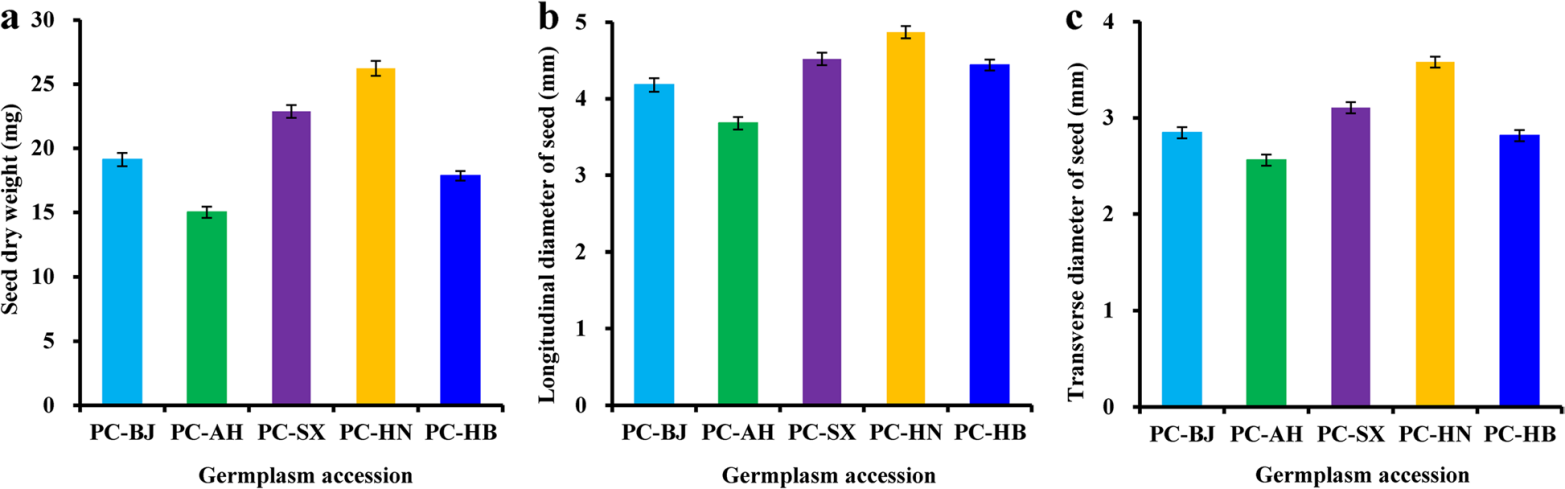
Variabilities on seed size and weight of 5 selected germplasm accessions of *P. chinensis*. **a** Variability on seed weight among different accessions. **b** Variability on seed longitudinal diameter across different accessions. **c** Variability on seed transverse diameter among different accessions. Error bars are standard deviations (SD) of three biological replicates, and the statistical results are presented in Additional file 1: Table S1

To better develop the *P. chinensis* seed oils as novel potential biofuel feedstock, it was crucial for us to select superior germplasm resources with high quantity and quality of seed oils for gaining ideal economic benefit. To achieve this, five plus trees with high seed yield (germplasm accessions PC-BJ, PC-AH, PC-SX, PC-HN, and PC-HB) [19] were selected as the materials to analyze the variability in the seed size and weight. Here, the dry weights of mature seeds varied from 15.01 mg (PC-AH) to 26.23 mg (PC-HN), followed by 18.84 mg (PC-HB), 19.12 mg (PC-BJ) and 22.87 mg (PC-SX) (Fig. 1a and Additional file 1: Table S1), indicating a notable difference in seed weight across different accessions. This allowed us to explore the differences in longitudinal and transverse diameter of the seeds from different accessions, and a notable variation on longitudinal diameter (3.68–4.87 mm) and transverse diameter (2.56–3.58 mm) of the seeds was detected among different accessions (Fig. 1b, c and Additional file 1: Table S1). Of note, the maximum seed weight (26.23 mg) with the greatest longitudinal diameter (4.87 mm) and transverse diameter (3.58 mm) was noted for accession PC-HN, reflecting that PC-HN as ideal accession could be utilized potentially for the plantation.

### Variability in seed oil content and oil body amount of 5 selected accessions of* P. chinensis*

**Fig. 2:**
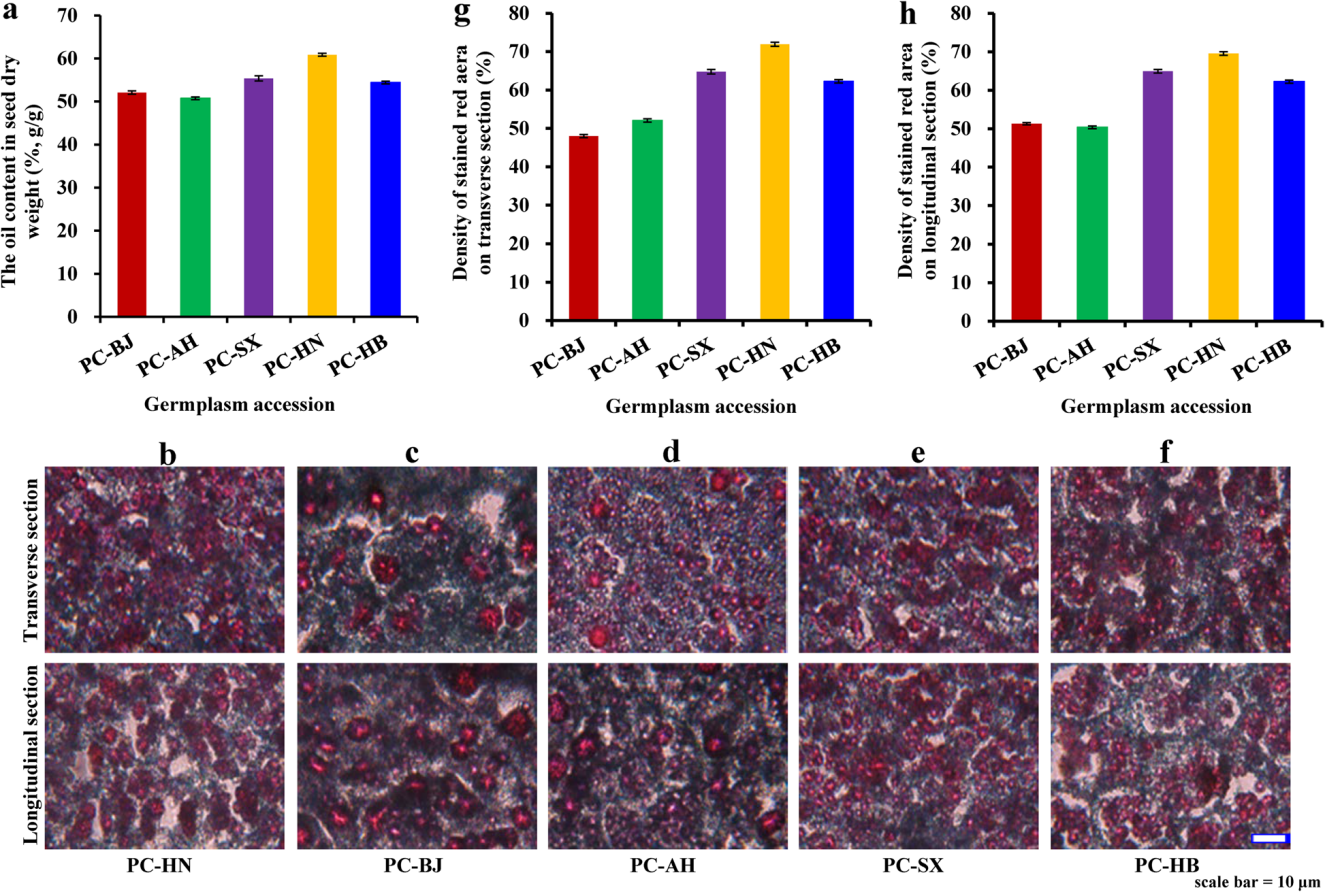
Variabilities on seed oil content and oil body amount of 5 selected germplasm accessions of *P. chinensis*. **a** Variability on seed oil content from different accessions. **b** Observation of lipid droplet on both longitudinal and transverse sections of the seeds from accession PC-HN colored by safranin with fast green. **c** Observation of lipid droplet on both longitudinal and transverse sections of the seeds from accession PC-BJ colored by safranin with fast green. **d** Observation of lipid droplet on both longitudinal and transverse sections of the seeds from accession PC-AH colored by safranin with fast green. **e** Observation of lipid droplet on both longitudinal and transverse sections of the seeds from accession PC-SX colored by safranin with fast green. **f** Observation of lipid droplet on both longitudinal and transverse sections of the seeds from accession PC-HN colored by safranin with fast green.** g** Variability on the scanned density of lipid droplet in transverse section among different accessions. **h** Variability on the scanned density of lipid droplet in longitudinal section among different accessions. Error bars are standard deviations (SD) of three biological replicates, and the statistical results are presented in Additional file 1: Table S1

High-quality oil content is known as one vital determinant of whether oil plant can be suitable for biodiesel production. Here, the highest content of seed oils was detected for accession PC-HN (60.88%), followed by PC-SX (55.37%), PC-HB (54.42%), PC-BJ (52.09%), and PC-AH (50.76%) (Fig. 2a and Additional file 1: Table S1), pointing to a difference in seed oil content across 5 selected accessions. In consideration of oil stored as a form of oil body, it was important to explore the relationship between oil content and oil body amount in the seeds of different accessions. In this work, the morphological difference of seed oil bodies across different accessions was observed by histochemical staining method. Of these, the larger and denser red-stained areas distributed on both longitudinal and transverse sections of the seeds were observed for accession PC-HN (Fig. 2b) compared with the seeds of other accessions (Fig. 2c-f), and the density of stained red area of seed oil bodies of PC-HN was also higher than that of other accessions (Fig. 2g, h and Additional file 1: Table S1), and thus concluded that the seeds of accession PC-HN contained more numbers of oil body cells required for oil storage, which was in line with the results of seed oil contents of different accessions (Fig. 2a).

### Variability in FA profiles of the seeds among 5 selected accessions of* P. chinensis*

Ideal FA compositions (FAs) of plant seed oils is vital for producing high-quality biodiesel, and thus FA compounds and its content of seed oils were analyzed across 5 selected accessions. Here, 7 kinds of FAs were identified in seed oils of all accessions (Table 1), including palmitic acid (C16:0), palmitoleic acid (C16:1), stearic acid (C18:0), oleic acid (C18:1), linoleic acid (C18:2), linolenic acid (C18:3) and arachidic acid (C20:0), of which the richest composition was oleic acid (42.05–69.94%) with an average of 51.97%, followed by linoleic acid (17.65–38.94%), palmitic acid (9.10–12.44%), and linolenic acid (1.13–4.41%), but the others exhibited small amount (0.24–2.11%). Of note, the maximum value of C18:1 (69.94%) and minimum level of C18:2 (17.65%) and C18:3 (1.13%) were all detected for accession PC-HN. Additionally, the total content of C18:1 and C18:2 in seed oils varied from 80.99% to 87.59%, and two accessions (PC-HN and PC-SX) had C18:1 content of more than 55% (Table 1), emphasizing that they were ideal raw material for biodiesel.

**Table 1:** Variabilities on the content of FA compositions in seed oil from *Pistacia chinensis* of different accessions

Accessions	FA compositions (%)^a^							Level of saturated and unsaturated FAs (%)		
	**C16:0**	**C16:1**	**C18:0**	**C18:1**	**C18:2**	**C18:3**	**C20:0**	**SFA**^**b**^	**MUFA**^**b**^	**PUFA**^**b**^
PC-BJ	12.06 ± 0.45	0.62 ± 0.02	2.11 ± 0.13	47.13 ± 1.15	34.15 ± 0.78	3.56 ± 0.17	0.37 ± 0.02	14.54 ± 0.61	47.75 ± 0.78	37.71 ± 0.48
PC-AH	11.55 ± 0.43	0.75 ± 0.04	1.95 ± 0.12	42.05 ± 1.21	38.94 ± 1.05	4.41 ± 0.12	0.35 ± 0.01	13.85 ± 0.45	42.80 ± 0.63	43.35 ± 0.72
PC-SX	9.55 ± 0.31	0.90 ± 0.03	1.05 ± 0.09	55.32 ± 1.64	30.94 ± 0.65	1.94 ± 0.07	0.29 ± 0.02	10.89 ± 0.29	56.22 ± 0.83	32.89 ± 0.51
PC-HN	9.10 ± 0.24	0.78 ± 0.02	1.16 ± 0.07	69.94 ± 2.02	17.65 ± 0.75	1.13 ± 0.04	0.24 ± 0.03	10.50 ± 0.35	70.72 ± 1.15	18.78 ± 0.38
PC-HB	12.44 ± 0.19	0.75 ± 0.04	1.67 ± 0.40	45.42 ± 1.24	36.43 ± 1.21	3.02 ± 0.03	0.28 ± 0.02	14.39 ± 0.41	46.17 ± 0.69	39.44 ± 0.64

^a^ Error bars are standard deviations (SD) of three biological replicates

^b^ SFA Saturated fatty acid (FA), MUFA Monounsaturated FA, PUFA Polyunsaturated FA

It is worth noting that raw plant oils required for ideal biodiesel production should contain a small level (< 30%) of saturated FA (SFA), high content (> 30%) of monounsaturated FA (MUFA), and low amount (< 60%) of polyunsaturated FA (PUFA) [12, 31]. In this work, the contents of MUFA and PUFA were detected for all accessions with the values of 42.80–70.72% and 18.78–43.35%, respectively, but the SFA content of all tested accessions was less than 15% (Table 1). Of these, PC-HN had the maximum content of MUFA (70.72%) and the minimum amounts of PUFA (18.78%) and SFA (10.05%), revealing that the seed oils from accession PC-HN with ideal FA profiles could satisfy the demand of superior biodiesel production.

**Fig. 3:**
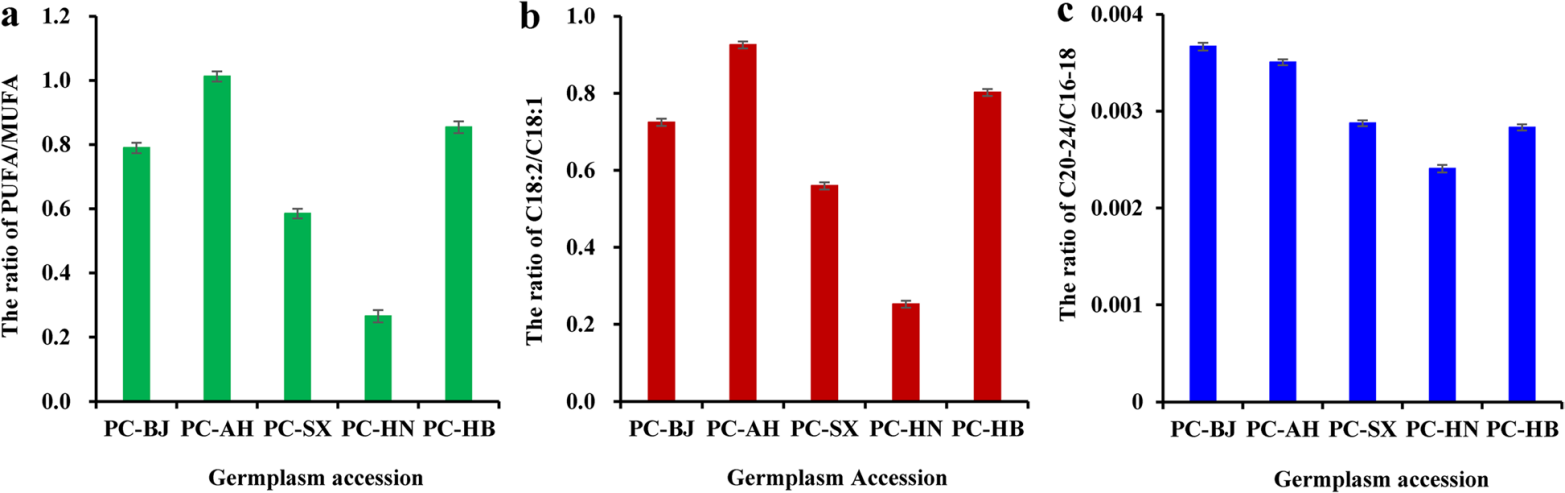
Variabilities on the ratios of PUFA/MUFA and C20-24/C16-18 in seed oils of *P. chinensis* from different germplasm accessions. **a** Variability of the PUFA/MUFA ratio in seed oils from different accessions. **b** Variability of C18:2/C18:1 ratio in the seed oils from different accessions. **c** Variability of C20-24/C16-18 ratio in the seed oils from different accessions. Error bars are standard deviations (SD) of three biological replicates, and the statistical results are presented in Additional file 2: Table S2

Another concern about low ratio of PUFA/MUFA (especially C18:2/C18:1) or C20-24/C16-18 as a critical index for assessing tribological properties of plant oils for utilization [32]. The ratios of PUFA/MUFA (0.265–1.013), C18:2/C18:1 (0.252–0.926) and C20-24/C16-18 (0.002–0.004) varied among different accessions (Fig. 3a-c and Additional file 2: Table S2), of which the minimum ratios of PUFA/MUFA (0.265), C18:2/C18:1 (0.252) and C20-24/C16-18 (0.002) were all recorded for accession PC-HN, indicating a good tribological property for the seed oils from accession PC-HN.

### Evaluation of biodiesel yield and fuel properties from seed oils among different accessions of* P. chinensis*

Considering a notable variation on oil content and total proportion of C18:1 and C18:2 in *P. chinensis* seeds of different accessions (Table 1 and Fig. 2a), it was essential to assess yield and fuel property of biodiesel derived from seed oils of different accessions for determining ideal accession as biodiesel feedstock. Here, the biodiesel yield from seed oils varied across different accessions, ranging from 84.78% (PC-AH) to 98.15% (PC-HN) with an average value of 92.17% (Table 2), of which biodiesel yield of PC-SX (96.76%) and PC-HN (98.15%) was in the standard of EN 14,214 (96.5%).

**Table 2:** Evaluation of biodiesel fuel properties of seed oils of *Pistacia chinensis* from different accessions

Accession or standard	Biodiesel fuel properties ^a^									
	**DU**	**LCSF**	**CN**	**IV (g/100 g)**	**CFPP (°C)**	**CP (°C)**	**OS (h)**	**KV (mm**^**2**^**/ s)**	**D (kg/m**^**3**^**)**	**Biodiesel yield (%)**^**b**^
Accession										
PC-BJ	126.74 ± 1.72	4.68 ± 0.25	49.77 ± 0.60	109.74 ± 1.11	-6.56 ± 0.17	-3.98 ± 0.12	2.90 ± 0.05	4.27 ± 0.08	878.00 ± 0.86	89.15 ± 1.07
PC-AH	133.91 ± 1.88	4.45 ± 0.22	48.91 ± 0.57	114.53 ± 1.28	-6.95 ± 0.22	-4.37 ± 0.11	2.63 ± 0.06	4.21 ± 0.09	878.84 ± 0.75	84.78 ± 1.03
PC-SX	123.95 ± 1.75	3.42 ± 0.19	50.11 ± 0.64	107.04 ± 1.05	-8.69 ± 0.22	-6.08 ± 0.09	3.01 ± 0.05	4.31 ± 0.08	877.66 ± 0.78	96.76 ± 1.13
PC-HN	109.40 ± 1.12	3.35 ± 0.20	51.87 ± 0.66	98.15 ± 1.04	-8.90 ± 0.27	-6.28 ± 0.11	3.57 ± 0.06	4.43 ± 0.10	875.89 ± 0.71	98.15 ± 1.05
PC-HB	128.06 ± 1.68	4.54 ± 0.27	49.61 ± 0.59	110.62 ± 1.22	-6.72 ± 0.21	-4.14 ± 0.08	2.85 ± 0.06	4.25 ± 0.09	878.16 ± 0.80	92.25 ± 1.05
Standard										
ASTM D6751	–	–	> 47	–	–	-3∼-12	≥ 3	1.9∼6.0	–	–
EN 14,214	–	–	> 51	≤ 120	< 5	–	≥ 6	3.5∼5.0	860∼900	96.5
GB/T 20,828	–	–	> 49	–	Report	–	≥ 6	1.9∼6.0	820∼900	–

^a^*DU* Degree of unsaturation, *LCSF *Chain length saturated factor, *CN* Cetane number, *IV* Iodine value, *CFPP* cold filter plugging point, *CP* cloud point, *OS* oxidation stability, *KV* kinematic viscosity, *D* density. Error bars are standard deviations (SD) of three biological replicates

^b^The biodiesel yield was expressed as the percentage (%, g/g) of the obtained total amount of FA methyl esters (g) to the used amount of raw oils (g)

Biodiesel properties, including iodine value (IV), cetane number (CN), oxidation stability (OS), cold filter plugging point (CFPP), cloud point (CP), density (D), kinematic viscosity (KV), chain length saturated factor (LCSF), and degree of unsaturation (DU), were evaluated with a difference across all tested accessions and described as the follows.

IV, one paramount structural parameter for estimating the unsaturated degree (DU) of FA and OS of biodiesel [33], varied from 98.15 to 114.53 in the FAMEs from seed oils of different accessions (Table 2), all of which were less than the specified maximum limit (120) of ASTM D6751, EN 14,214 and GB/T20,828 standards. Also, CN is one vital index for determining ignition delay time and combustion quality of biofuels, and its values of all accessions ranged from 48.91 to 51.87, all of which could satisfy the standard of USA (ASTM D6751: CN > 47), and most accessions (except PC-AH) was also in concordance with China standard (GB/T 20,828: CN > 49), while only PC-HN (51.87) could meet Europe standard (EN 14,214: 51 < CN < 65) (Table 2), indicating that the biodiesels from seed oils of all accessions had ideal ignition quality.

KV is one key criteria for determining flow ability of biodiesel, and spray penetration and atomization of fuel [33]. The range of KV value (4.21–4.43 mm^2^/s) of all accessions could meet the standards of EN 14,214 (3.5 < KV < 5.0), ASTM D6751 (1.9 < KV < 6.0), and GB/T20828 (1.9 < KV < 6.0) (Table 2), referring a good flow or spray ability of biodiesel fuel from seed oils of all accessions. Also, density (*D*), one key index for assessing combustion efficiency of biodiesel [34], ranged from 875.89 to 878.84 kg/m^3^ of all accessions (Table 2), could satisfy the standards of GB/T20,828 (820 < D < 900) and EN 14,214 (860 < D < 900), implying ideal combustion efficiency for the biodiesels from *P. chinensis* seed oils of all accessions.

OS is one vital technical parameter relevant for stability of biodiesel reaction with air [33]. Our detected OS values of biodiesel fuel from the seed oils of all accessions varied from 2.63 to 3.57 h (Table 2), all of which did not reach the minimum limit (6 h) specified in the standards of EN 14,214 and GB/T 20,828, but only accession PC-HN (3.57 h) could meet the ASTM D6751 standard (OS > 3.0 h), which may be attributable to low amount of PUFA (18.78%) in seed oils of PC-HN compared with other accessions (34.59–44.25%) (Table 1).

Both CFPP and CP are known as two key low-temperature parameters to determine the maximum of filterability, but not limited by the US and European standards. The CFPP valve ranged from -8.90 °C to -6.56 °C for biodiesels across all accessions (Table 2), less than the maximum limit (0 °C) of Germany standard (DIN V51,606) in summer, but higher than the minimum limit (-10.0 °C) for spring and autumn. Also, all accessions had an ideal CP value (-6.28 °C to -3.98 °C) (Table 2) within ASTM D6751 standard (-12 °C < CP < -3 °C), implying a good cold flow property of biodiesel from seed oils of all accessions, especially PC-HN with the minimum value of CFPP (-8.90 °C) and CP (-6.28 °C), which was attributed to high level of unsaturated FA (Fig. 3a) and small amount of length chain saturated factor (LCSF) (Table 2).

Also noteworthy was the C18:3 content and the FAs with four double bonds in FAMEs. Low amount (1.13–4.41%) of C18:3 in seed oils of all accessions and no four double-bond FAs (C18:4 and C20:4) in the FAMEs (Table 1) all met the EN14,214-2008 specification (< 12% and 1%, respectively).

Another concern about DU and LCSF as two additional parameters based on the type/structure of FAs. The values of CN, IV and OS are affected greatly by the DU [31], but both CFPP and CP mostly depends on LCSF [31, 32]. The DU values were detected in the range of 109.40 to 133.91 across all accessions, of which PC-HN had a minimum of 109.40, with the highest values of CN (51.87) and OS (3.57 h) and the lowest value of IV (98.15) (Table 2). However, a negative correlation of LCSF with CFPP or CP was noted across all accessions (Table 2). It seems therefore that both DU and LCSF may be as vital indicators for evaluating biodiesel fuel properties of seed oils of *P. chinensis* from different accessions.

### Global identification of potential TFs specific for high oil accumulation in *P*. *chinensis* seeds

**Fig. 4:**
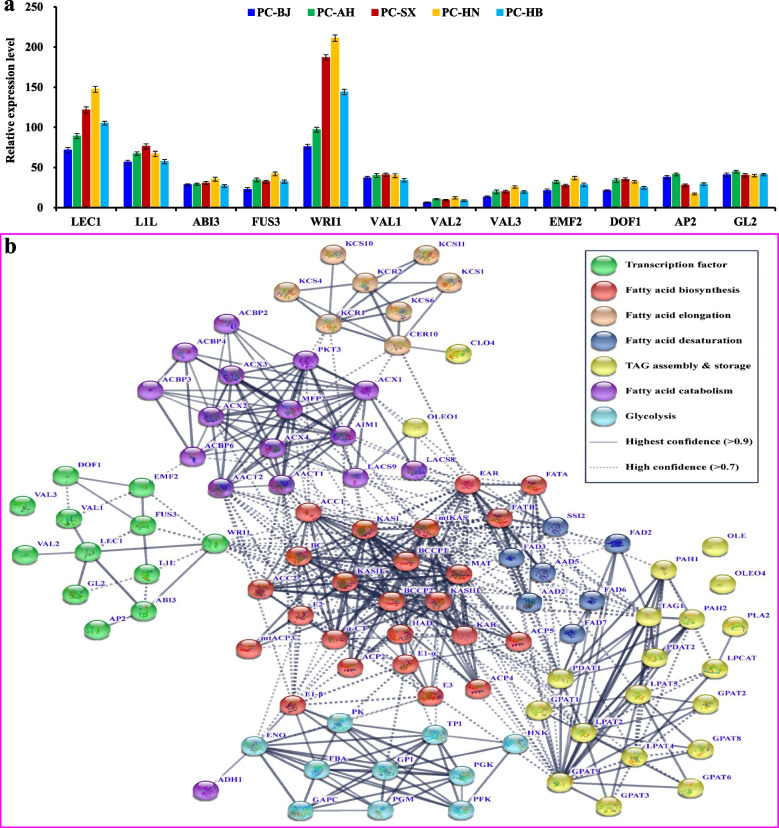
Analyses of transcript differences of transcription factors (TFs) in *P. chinensis* seeds of different accessions and protein interaction between the TFs and oil-synthesized enzymes. **a** Analysis of transcript differences of the TFs in the seeds across different accessions by qRT-PCR. The genes encoding for ubiquitin-conjugating enzyme (*UBC*) and large subunit ribosomal protein L32e (*RPL32e*) were used as internal controls, and its expression level was arbitrarily set to 1.00 for standardization. Error bars are SD of three biological replicates with three technical repetitions each, and the statistical results are shown in Additional file 3: Table S3. **b** A protein interaction network was constructed for the TFs and oil-synthesized enzymes or transporters by using STRING software with high network connectivity (confidence score > 0.7)

Another one interesting question was the mechanism that govern such difference in seed oil content and FA profile of *P. chinensis* across all accessions (Table 1 and Fig. 2a). FA synthesis and oil accumulation of plants are highly controlled by the TFs, but the question of which TF and how to regulate seed oil accumulation of *P. chinensis* is still elusive. Recently, differential transcripts were annotated for 12 TFs (LEC1, ABI3, L1L, FUS3, WRI1, VAL1, GL2, AP2, VAL2, VAL3, EMF2 and DOF1) involving potentially in seed oil synthesis of *P. chinensis* by our transcriptome assay (SRP030781) [17], which promoted to unravel the potential association of their transcript levels with accumulative amount of seed oils across all accessions with the aim to identify TFs essential for high oil production of *P. chinensis* seeds. Here, transcript levels of WRI1 and LEC1 were much higher than those of the other 9 TFs (Fig. 4a and Additional file 3: Table S3), and importantly, only both LEC1 and WRI1 transcripts exhibited a pattern that highly correlated with seed oil content across all accessions (Figs. 2a and 4a). Hence, we further performed protein interaction assay for our all identified TFs as an important attempt to explore TF-mediated regulation mechanism of high oil production of the seeds, in which some enzymes crucial for oil accumulation, including carbon source provision (PK, GPI, ENO, E1-α, E1-β, and E3), de novo FA biosynthesis and elongation (ACC2, BCCP1/2, KASI/III, EAR, MAT and FATA/B), FA desaturation (FAD2/3), and TAG assembly and storage (TAG1, PDAT1 and GPAT9), were identified to be highly correlated with WRI1 (interaction score > 0.7), indicating a central regulator of WRI1 for oil accumulation in *P. chinensis* seeds. Notably, LEC1 displayed the highest confidence level (interaction score = 0.958) to interact with WRI1 among the selected TFs (Fig. 4b), and thus considered that LEC1 and its targeted WRI1, located in center position of interaction network, may contribute mostly to regulate transcriptions of enzymes in relation to oil accumulation of *P. chinensis* seeds.

Together, accession PC-HN with high seed oil content and biodiesel yield, and ideal fuel property, could be suitable for high-quality biodiesel production, and LEC1/WRI1-mediated transcription regulatory network may play a major role in FA synthesis and oil accumulation in *P. chinensis* seeds of different accessions. Hence, our following work focused on identifying the functions of WRI1 and LEC1 and exploring their molecular regulatory mechanism in *P. chinensis* seeds.

### Isolation and structural analysis of *PcWRI1* and *PcLEC1* genes from *P. chinensis* seeds

**Fig. 5:**
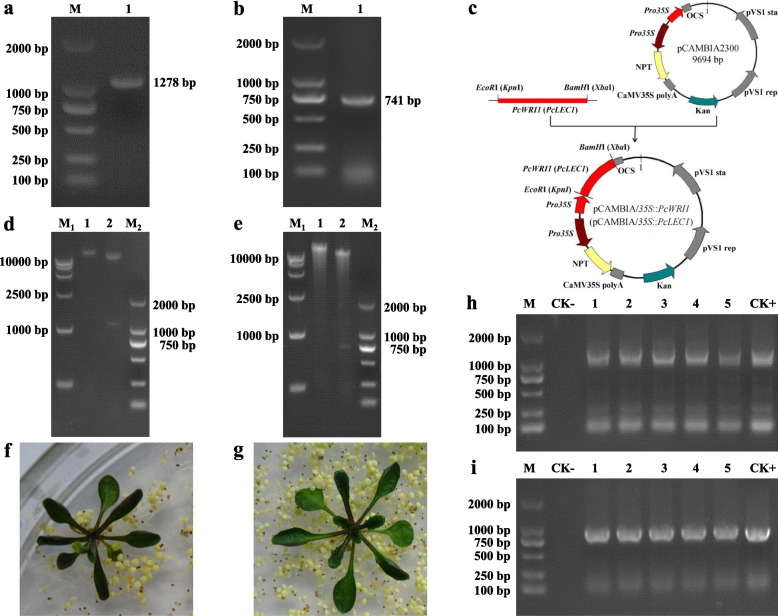
Isolation of *PcWRI1* and *PcLEC1* genes and genetic transformation of Arabidopsis. **a** Cloning of *PcWRI1* gene from *P. chinensis* seeds by PCR (M: DNA marker; 1: *PcWRI1* gene fragment). **b** Cloning of *PcLEC1* gene from *P. chinensis* seeds by PCR (M: DNA marker; 1: *PcLEC1* gene). **c** Illustration of binary vector of pCAMBIA/*35S*::*PcWRI1* or pCAMBIA/*35S*::*PcLEC1*. **d** Verification of pCAMBIA/*35S*::*PcWRI1* construction by restriction enzyme-digested assay (M_1_: DNA marker; 1: single enzyme-digested product by *EcoR* I; 2: double enzyme-digested product by *EcoR* I and *BamH* I; M_2_: DNA marker). **e** Verification of pCAMBIA/*35S*::*PcLEC1* construction by restriction enzyme-digested assay (M_1_: DNA marker; 1: single enzyme-digested product by *Xba* I; 2: double enzyme-digested product by *Xba* I and *Kpn* I; M_2_: DNA marker). **f** Resistance screening of *PcWRI1* transgenic Arabidopsis. **g** Resistance screening of *PcLEC1* transgenic Arabidopsis. **h** Verification of integration of *PcWRI1* within genome of Arabidopsis lines by PCR detection (M: DNA marker; CK-: WT negative control; 1–5: *PcWRI1* transgenic lines; CK + : vector positive control). **i** Verification of integration of *PcLEC1* within genome of Arabidopsis by PCR assay (M: DNA marker; CK-: WT negative control; 1–5: *PcLEC1* transgenic lines; CK + : vector positive control). The full-length gels are presented in Additional file 4: Fig. S1

To gain a better insight into LEC1/WRI1-mediated regulatory mechanism for high oil accumulation of *P. chinensis* seeds, based on our recent transcriptome sequencing results from *P. chinensis* seeds [17], the full-length cDNA sequences of *WRI1* (1,377 bp) and *LEC1* (988 bp) (designated as *PcWRI1* and *PcLEC1*, respectively) were obtained from the seeds of ideal accession PC-HN, with the open reading frames (ORFs) of 1,278 bp and 741 bp, encoding proteins containing 425 and 247 amino acids, respectively (Fig. 5a, b and Additional file 4: Fig. S1).

To predict the potential biological functions and evolutionary relationships of *PcWRI1* and *PcLEC1*, the assays were conducted for phylogenetic tree, multiple sequence alignment, conserved domain and 3D structure. Here, PcWRI1 and PcLEC1 had typical ‘VYL’ and ‘CBFD_NFYB_HMF’ motifs respectively, and showed high-similar amino acid sequence and 3D structure with other oilseed plants (such as *Glycine max* and *Zea mays*) (Additional file 5: Fig. S2), pointing to a semblable regulatory function of PcWRI1 and PcLEC1 in FA biosynthesis.

To determine the function of *PcWRI1* or *PcLEC1* from *P. chinensis* seeds, the CaMV35S promoter-driven expressing vectors of *PcWRI1* (*CaMV35S::PcWRI1*) and *PcLEC1* (*CaMV35S::PcLEC1*) were constructed and transformed into model plant Arabidopsis respectively. The T3 homozygous seeds were obtained by resistance selection, PCR assay (Fig. 5c-i) and chi-square (*χ*^2^) test (Additional file 6: Table S4), from which T4 transgenic plants were used for further analyses.

### Morphological analyses of transgenic *PcWRI1 and PcLEC1* lines

**Fig. 6:**
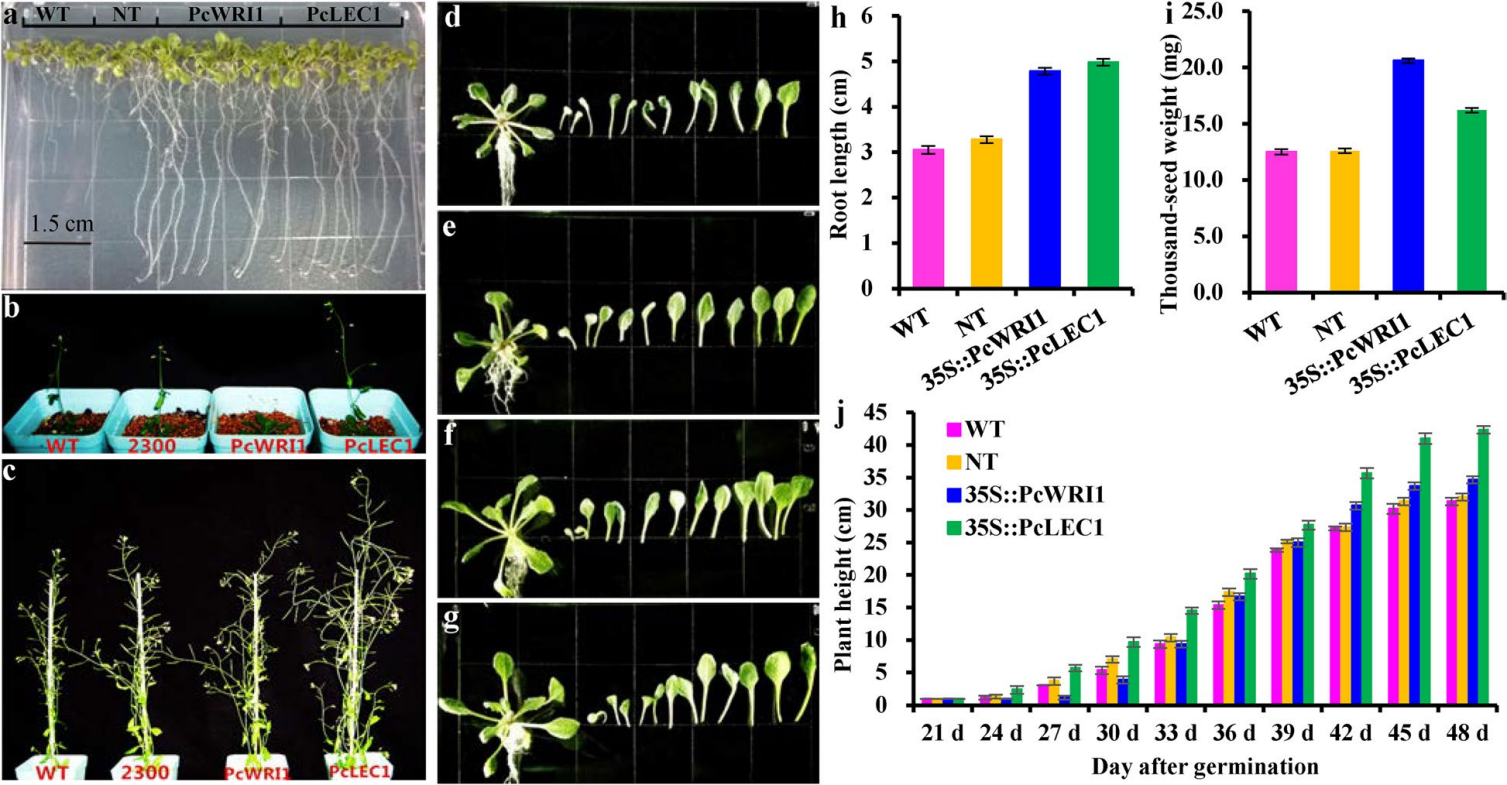
Phenotype analyses among the controls (WT and NT) and *PcWRI1* or *PcLEC1* transgenic lines of Arabidopsis. **a** The root growth of different transgenic Arabidopsis germinated on half-strength MS medium for 10 d. **b** Growth status of different transgenic lines at 27 d after germination. **c** Growth status of different transgenic lines at 48 d after germination. **d** The size of rosette leaves of the WT lines at 21 d. **e** Size of rosette leaves of the NT lines at 21 d. **f** Size of rosette leaves of *PcWRI1* transgenic lines at 21 d. **g** Size of rosette leaves of *PcLEC1* transgenic lines at 21 d. **h** Detection of root length of T3 seed germinated on half-strength MS medium for 10 d. **i** Dry weight of thousand mature seeds from different transgenic lines. **j** Analyses of plant heights of different transgenic lines at 21–48 d. The wild type (WT) and null transgenic lines (NT) were used as the controls. The error bars are standard deviations (SD) of three biological replicates, and the statistical results are presented in Additional file 7: Table S5

To examine whether ectopic overexpression of *PcWRI1* or *PcLEC1* affected morphological changes in transgenic lines, a concurrent exploration was conducted on root length, plant height, rosette and leaf size, and seed dry weight. After germination of T3 seeds on half-strength MS medium for 10 d, the root lengths of *PcLEC1* and *PcWRI1* transgenic lines were detected with 4.98 and 4.75 cm, respectively, which were all longer than those of wild type (WT, 3.05 cm) and null transgenic lines (NT, 3.28 cm) (Fig. 6a, h). *PcLEC1* transgenic line had the earliest bolting time (Fig. 6b), about 3 and 5 d earlier than that of the control and *PcWRI1* transgenic lines, respectively, and its mature plant height (42.33 cm) was higher than *PcWRI* transgenic plants (34.67 cm), both of which were higher than that of WT (31.33 cm) and NT (30.04 cm) (Fig. 6c, j). Compared with the controls (WT and NT), *PcWRI1* and *PcLEC1* transgenic lines could produce the larger and longer rosette radius and leaf, but the number of rosette leaves showed no significant change among all tested lines (Fig. 6d-g). These results implied that ectopic overexpression of *PcWRI1* (especially *PcLEC1*) could promote growth and development of root, stem and leaf of transgenic plants. Also, the dry weights of mature 1,000 seeds from transgenic *PcWRI1* and *PcLEC1* lines were 20.60 mg and 16.21 mg, respectively, about 0.65- and 0.30-fold higher than those of the controls (Fig. 6i and Additional file 7: Table S5), emphasizing that ectopic overexpression of *PcLEC1* (especially *PcWRI1*) could facilitate reproductive development and seed growth of transgenic lines.

### Effect of *PcWRI1* or* PcLEC1* overexpression on seed oil accumulation and biodiesel fuel property

**Fig. 7:**
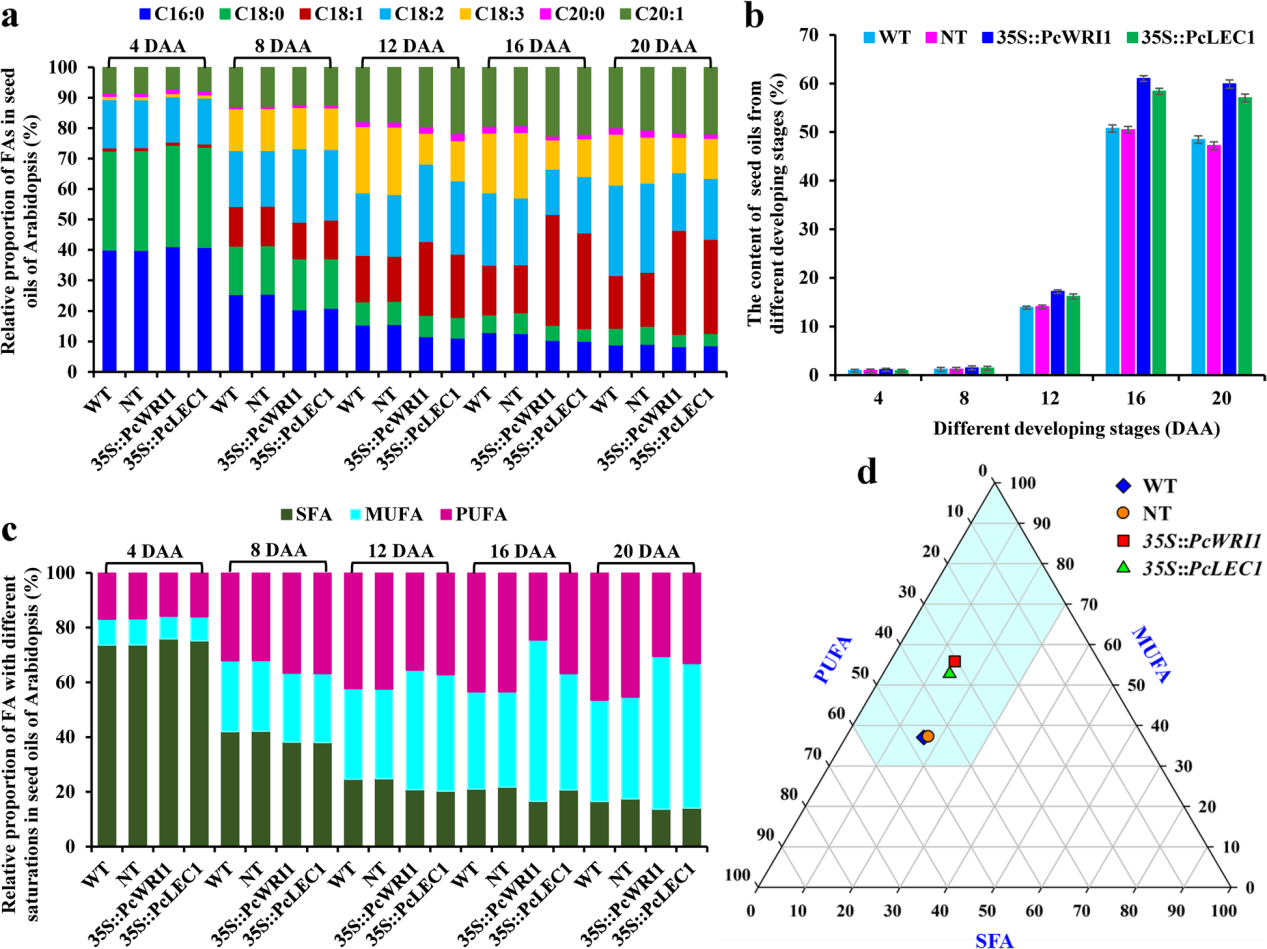
Dynamic changes of oil content and FA compositions in developing seeds of *PcWRI1* or *PcLEC1* in transgenic Arabidopsis. **a** Dynamic changes of fatty acid (FA) compositions in seed oils of the controls (WT and NT) and *PcWRI1* or *PcLEC1* in transgenic Arabidopsis during development. **b** Dynamic changes of oil content in developing seeds from the controls (WT and NT) and *PcWRI1* or *PcLEC1* in transgenic Arabidopsis. **c** Dynamic changes of relative proportion of monounsaturated, polyunsaturated and saturated FAs in developing seeds of the controls (WT and NT) and *PcWRI1* or *PcLEC1* in transgenic Arabidopsis. **d** Construction of prediction model for biodiesel fuel property of raw ripen seed oils from the controls (WT and NT) and *PcWRI1* or *PcLEC1* in transgenic Arabidopsis. The light-blue part of the region was clearly delineated to predict biodiesel fuel properties that could fully meet the limit of cetane number, iodine number, cold filter plugging point and oxidation stability. Error bars are standard deviations (SD) of three biological replicates, and the statistical results are presented in Additional file 8: Table S6

To explore potential effect of *PcWRI1* or *PcLEC1* overexpression on oil accumulation of transgenic seeds, we detected dynamic patterns of oil content and FA compositions during seed development of WT, NT, and transgenic *PcWRI1* and *PcLEC1* lines by GC–MS. Here, the oil contents of developing seeds of all tested lines gradually increased at 4–16 d after anthesis (DAA) with a rapid increase at 12–16 DAA, followed by a slight decrease at 20 DAA (full rape) (Fig. 7b and Additional file 8: Table S6). Of note, the oil contents of ripen dry seeds of *35S*::*PcWRI1* and *35S*::*PcLEC1* lines were 59.88% and 57.04%, respectively, both of which was higher than that of the WT (48.45%) and NT (47.19%), implying that overexpression of *PcWRI1* or *PcLEC1* increased seed oil content, but *PcWRI1* overexpression showed the relatively great effect on seed oil accumulation.

A total of 7 kinds of FAs were identified in developing seed oils of all tested lines (*35S*::*PcWRI1*, *35S*::*PcLEC1*, NT, and WT), including C16:0, C18:0, C18:1, C18:2, C18:3, C20:0 and C20:1 (Fig. 7a), all of which displayed no significant difference in oil content of developing seeds at 4–8 DAA across all lines. The contents of C16:0 and C18:0 decreased in the seeds of all tested lines during whole development (4–20 DAA), but an obvious decrease was noted for transgenic seeds. The contents of C18:1, C18:2 and C20:1 in seed oils of both NT and WT gradually increased at 4–20 DAA, but an increase of these was recorded for transgenic seeds before 16, 12 and 16 DAA, respectively. Also, a notable increase of C18:3 content in the controls (NT and WT) and transgenic seeds was observed before 16 and 8 DAA, respectively. Given that a higher amount of C18:1 and C20:1 and a lower content of other FAs (C16:0, C18:0, C18:2 and C18:3) were detected for ripen seeds of transgenic lines compared with the control, it seems likely that overexpression of *PcWRI1* or *PcLEC1* could result in an alteration of FA profile in seed oils of transgenic lines.

Another concern was that whether the overexpression of *PcWRI1* or *PcLEC1* was implicated in affecting biodiesel fuel property of seed oils from transgenic lines. To this end, the percentages of SFA, MUFA and PUFA in ripen seed oils of all tested lines (Fig. 7c) were calculated as three angular points to establish a triangular graph. It was found that all tested lines were presented in light-blue area of our constructed prediction model, but *P****c****WRI1* and *PcLEC1* transgenic lines was located at the far end of PUFA angular point (lower left vertex) and SFA angular point (lower right vertex) (Fig. 7d), implying that seed oils from transgenic lines had good fuel properties (CN, IN, CFPP, OS and CP) [12, 16]. Thus, *PcWRI1* or *PcLEC1* overexpression could improve biodiesel property of seed oils, which coincided with the fact that a higher MUFA mount (55.83% and 52.76%, respectively) and a lower PUFA level (30.52% and 33.15%, respectively) were detected for ripen seed oils of transgenic *PcWRI1* and *PcLEC1* lines compared with the WT (37.04% and 46.46%, respectively) (Fig. 7c).

### Analyses of spatiotemporal expressions of *PcWRI1* and *PcLEC1* in transgenic lines

**Fig. 8:**
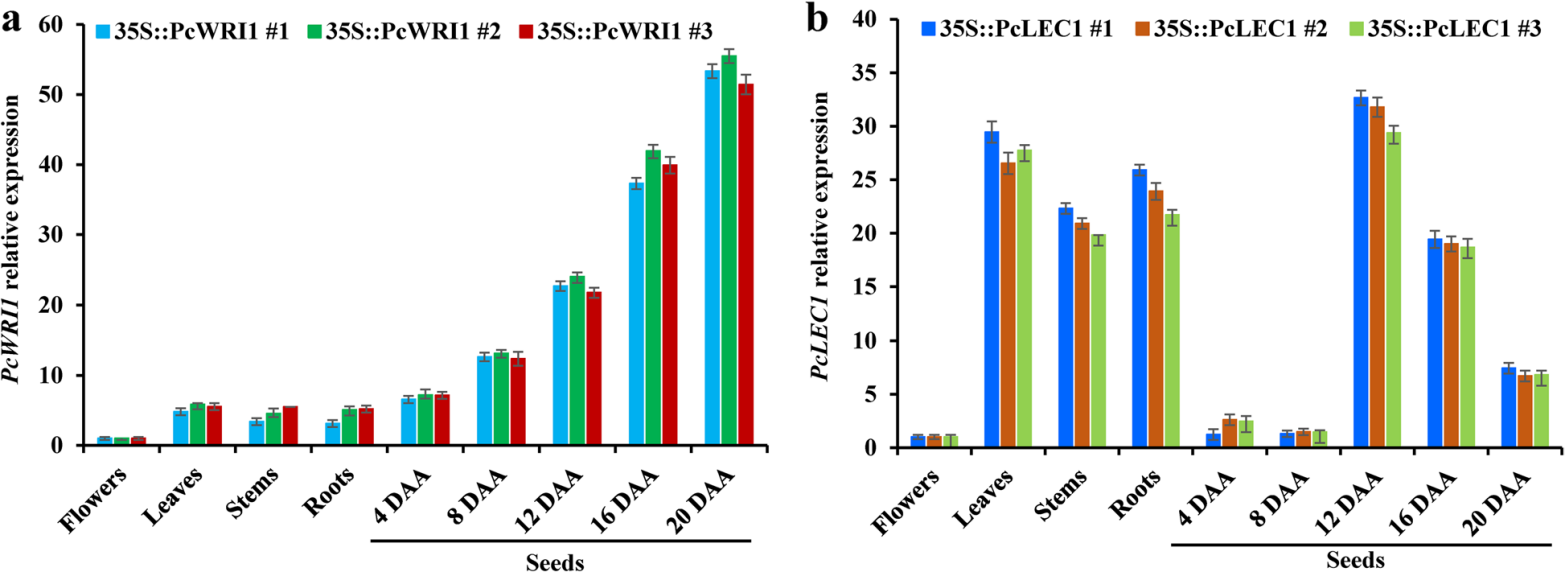
Analyses of spatiotemporal expressions of *PcWRI1* and *PcLEC1* in different tissues and developing seeds of transgenic Arabidopsis by qRT-PCR. **a** Differential transcript patterns of *PcWRI1* in different tissues and developing seeds of transgenic lines. **b** Differential transcript patterns of *PcLEC1* in different tissues and developing seeds of transgenic lines. The expression level from flower sample was arbitrarily set to 1.00 for standardization, and Arabidopsis *ACTIN* gene was used as internal control. Error bars are SD of three biological replicates with three technical repetitions each, and the statistical results are presented in Additional file 9: Table S7

To gain insight into molecular regulation mechanism of *PcWRI1* and *PcLEC1* on FA synthesis and oil accumulation, we explored spatial–temporal expression pattern of *PcWRI1* or *PcLEC1* in three T3 independent transgenic lines (Fig. 8 and Additional file 9: Table S7). Here, low transcript level of *PcWRI1* was identified in vegetative organs (flowers, leaves, stems and roots) of *PcWRI1* transgenic lines, but a gradual and significant increase was marked for the seeds during development (Fig. 8a), emphasizing that *PcWRI1* was specifically expressed in seed with development-dependently increase pattern. Yet, *PcLEC1* transcript was detected with high level in the leaves, stems and roots of *PcLEC1* transgenic lines, and a sharp peak in transcript was observed in developing seeds from 8 to 12 DAA, followed by a decrease (Fig. 8b). Also, transcript level of *PcLEC1* in the leaves, stems and roots was higher than that of *PcWRI1* in those vegetative organs of *PcWRI1* transgenic lines. These results revealed a difference in spatiotemporal expression pattern between *PcWRI1* and *PcLEC1*.

### Effect of ectopic *PcWRI1* or *PcLEC1* on transcript of oil synthesis-related TFs in developing seeds of transgenic lines

**Fig. 9:**
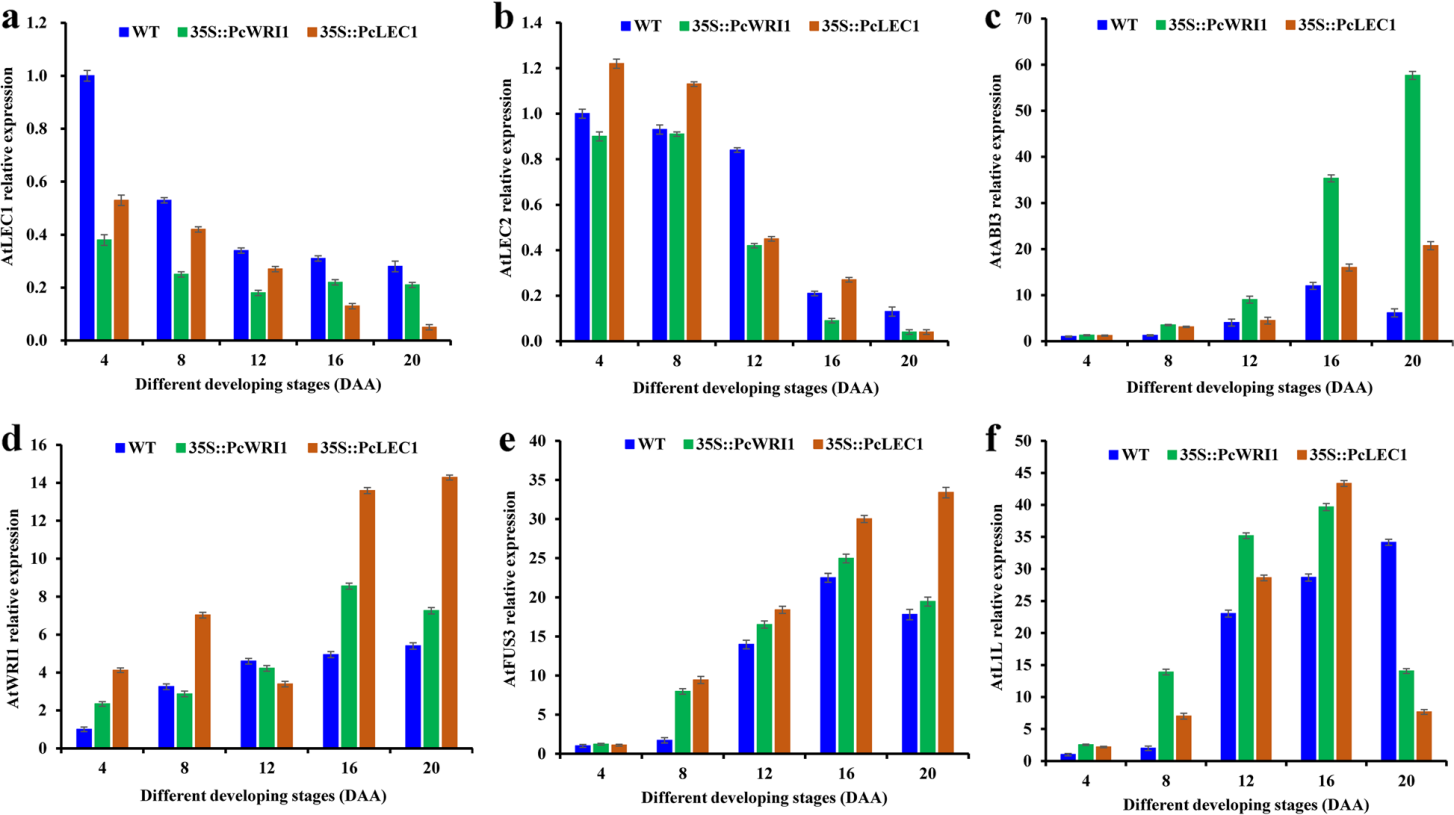
Effect of ectopic *PcWRI1* or *PcLEC1* on transcripts of oil biosynthesis-related TFs in developing seeds of transgenic Arabidopsis by qRT-PCR. **a** Comparative assay of temporal transcript patterns of endogenous *AtLEC1* in developing seeds of transgenic Arabidopsis with *PcWRI1* or *PcLEC1* overexpression. **b** Comparative assay of temporal transcript patterns of endogenous *AtLEC2* in developing seeds of *PcWRI1* or *PcLEC1* overexpression transgenic Arabidopsis. **c** Comparative assay of temporal transcript patterns of endogenous *AtABI3* in developing seeds of *PcWRI1* or *PcLEC1* overexpression transgenic Arabidopsis. **d** Comparative assay of temporal transcript patterns of endogenous *AtWRI1* in developing seeds of transgenic Arabidopsis with *PcWRI1* or *PcLEC1*. **e** Comparative analysis of temporal transcript patterns of endogenous *AtFUS3* in developing seeds of transgenic Arabidopsis with *PcWRI1* or *PcLEC1* overexpression. **f** Comparative assay of temporal transcript patterns of endogenous *AtL1L* in developing seeds of *PcWRI1* or *PcLEC1* overexpression transgenic Arabidopsis. Arabidopsis *ACTIN* gene was used as endogenous reference, and expression level from seed sample at 4 DAA was arbitrarily set to 1.00 for standardization. Error bars are SD of three biological replicates with three technical repetitions each, and the statistical results are presented in Additional file 10: Table S8

To further examine the mechanism required for an alteration in seed oil content in *PcWRI1* or *PcLEC1* over-expressing transgenic lines (Fig. 7b), it was vital to explore whether ectopic expression of *PcWRI1* or *PcLEC1* affected transcript of endogenous oil-related TFs (such as L1L, LEC2, FUS3 and ABI3) in the transgenic seeds during development (Fig. 9 and Additional file 10: Table S8). Compared with the WT, ectopic overexpression of *PcWRI1* or *PcLEC1* could result in a decrease in transcript level of endogenous *AtLEC1* and *AtLEC2* during seed development (Fig. 9a, b), while the notably upregulated transcript was noted for *AtABI3*, *AtWRI1*, *AtFUS3* and *AtL1L* (Fig. 9c-f), and thereby reflected that ectopic overexpression of *PcLEC1* could prompt transcript of its targets (*AtABI3*, *AtWRI1*, *AtFUS3* and *AtL1L*).

### Temporal transcripts of oil accumulation-related genes in developing seeds of *PcWRI1* or *PcLEC1* transgenic lines

To highlight how *PcWRI1* and *PcLEC1* regulate FA biosynthesis and oil accumulation at molecular level, the comparative analysis of temporal transcript changes was conducted on 28 key genes encoding for regulatory enzymes in developing seeds of the WT and transgenic lines, involved specifically in carbon source supply (glycolysis and acetyl-CoA formation) and oil synthetic process (FA synthesis and modification, and TAG assembly and oil storage).

**Fig. 10:**
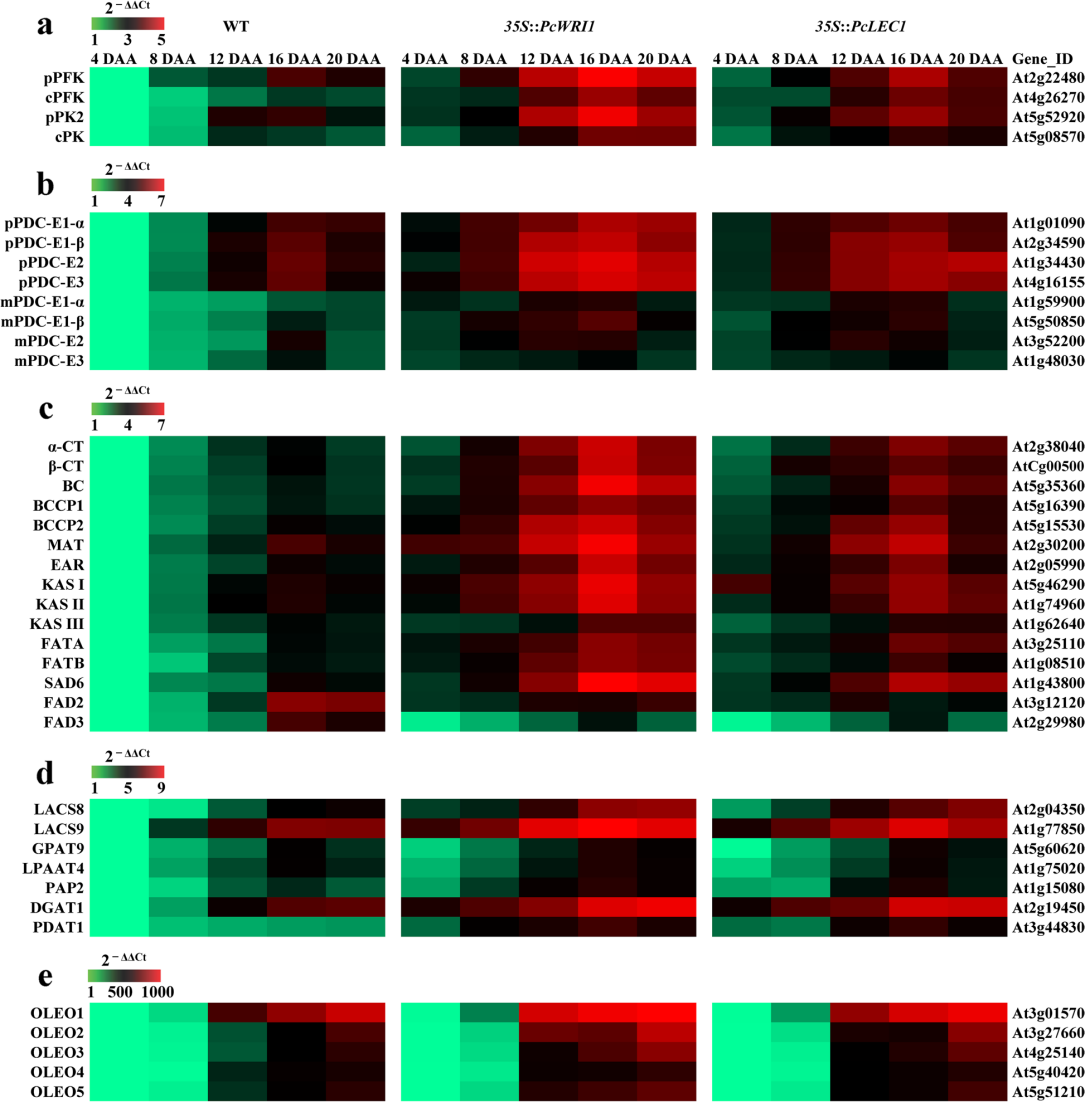
Temporal transcriptional assay for enzymes and proteins involved in oil accumulative process in developing seeds of *PcWRI1* or *PcLEC1* transgenic lines by qRT-PCR. **a** Differential transcripts for genes involved in glycolysis during seed development. **b** Differential transcripts for enzymes reluctant for acetyl-CoA formation during seed development. **c** Differential transcripts for enzymes in de novo FA synthesis during seed development. **d** Differential transcripts for enzymes involved in FA export and TAG assembly during seed development. **e** Differential transcripts for oil body proteins involved in oil body formation during seed development. Arabidopsis *ACTIN* gene was used as internal control. The relative expression values in heatmap were counted as 2-^△△Ct^, and the expression level from seed sample at 4 DAA was arbitrarily set to 1.00 for standardization. The cytosolic (c), plastidic (p) or mitochondrial (m) isoforms of the enzymes are indicated by a prefix in figure (a) and (b)

Acetyl-CoA, one key precursor for FA biosynthesis, is mostly derived from pyruvate (PYR), involved in glycolysis and PYR dehydrogenase complex (PDC). By qRT-PCR, the differentially up-regulated transcripts were marked for PYR kinase (PK) and phosphofructokinase (PFK) of plastidic and cytosolic glycolysis, and mitochondrial and plastidic PDC subunits (E1-α, E1-β, E2 and E3) relevant for acetyl-CoA formation during seed development of both the control and transgenic lines, of which plastidic PFK, PK and PDC all exhibited a higher transcript (Fig. 10a, b), emphasizing an importance of plastidic glycolysis and PDC in providing carbon sources (PYR and acetyl-CoA) for FA synthesis in developing seeds. Yet, abundant transcript of PFK, PK and PDC was all identified in developing seeds of transgenic *PcWRI1* lines as compared with transgenic seeds of *PcLEC1*, both of which were higher than that of the WT (Fig. 10a, b), indicating that *PcWRI1* or *PcLEC1* overexpression could strongly increase plastidic glycolysis capacity and PDC activity via effective transcriptional regulation to provide large acetyl-CoA destined for high FA production in developing seeds of transgenic lines.

High oil accumulation in oil plants is highly dependent on high FA synthesis and TAG assembly as well as oil storage, and thus we performed qRT-PCR to explore quantitative association of oil accumulative amount with oil-synthetic gene transcript level in developing seeds of all tested lines. Compared with the WT, overexpression of *PcLEC1* or *PcWRI1* significantly increased transcripts of FA synthetic enzymes in developing seeds, including acetyl-CoA carboxylase (ACC), 3-ketoacyl ACP synthase I/II/III (KAS I/II/III), fatty acyl-ACP thioesterase A/B (FATA/B), malonyl-CoA-ACP transferase (MAT), 18:0-ACP desaturase 6 (SAD6) and enoyl-ACP reductase (EAR), but a higher transcript level of them was noted for *PcWRI1* transgenic seeds (Fig. 10c), as was the case for key enzymes of TAG assembly [acyl-CoA:G3P acyltransferase 9 (GPAT9), acyl-CoA:LPA acyltransferase 4 (LPAAT4), PA phosphatase 2 (PAP2), DAG acyltransferase 1 (PDAT1) and acyl-CoA:DAG acyltransferase 1 (DGAT1)] (Fig. 10d) and TAG accumulation-related oil body protein [oleosin-1/-2/-3/-4/-5 (OLEO1/2/3/4/5)] (Fig. 10e). A temporal and strong correlation of their transcript levels with oil content of developing seeds of transgenic lines (Figs. 7b and 10c, d) reflected that *PcLEC1* overexpression (especially *PcWRI1*) could activate transcript of multiple genes required for FA synthesis, TAG assembly and oil storage in developing seeds of transgenic lines. Of note, transcript level of DGAT1 was much higher than that of PDAT1 across all tested lines (Fig. 10d), implying that DGAT1 may be a crucial regulator for TAG assembly, and the most abundant transcript of *OLEO1* implies (Fig. 10e) its importance in seed oil body formation. Also noteworthy was the role of long chain acyl-CoA synthetase (LACS) in the export of plastidic free FA to produce acyl-CoA pool for TAG formation [35]. High transcript of LACS8/9 (Fig. 10d) in developing seeds of transgenic lines was paralleled to that of FA-synthesized enzymes (Fig. 10c), and showed a pattern that correlated with oil amount during seed development (Fig. 7b), and thus referred that overexpression of *PcLEC1* or *PcWRI1* could promote LACS8/9 transcript to export FA from plastid into ER for acyl-CoA pool generation destined to TAG assembly.

Another concerned was that the PUFA (C18:2 and C18:3) level, as a key factor affecting the quality of plant oils [36], was produced from desaturation of C18:1 by FAD2 and FAD3, respectively. The differentially upregulated transcripts of FAD2 (high) and FAD3 (low) were detected in developing seeds of all lines, but the relative high transcript of them was identified for the WT (Fig. 10d), reflecting that *PcLEC1* or *PcWRI1* overexpression could suppress transcript of *FAD2/3*, causing high content of C18:1 and low amount of C18:2 and C18:3 in ripen seed oils of both transgenics lines (Fig. 7a).

## Discussion

### *P. chinensis* seed oil as novel and potential woody feedstock for biodiesel production in China

To determine ideal accession for developing *P. chinensis* seed oil as novel woody feedstock for biodiesel production, the cross-accessions comparisons were conducted on a subsequent evaluation of seed phenotypic characteristics (size and weight), oil body amount, oil content, FA profile, biodiesel yield and fuel properties in *P. chinensis* seeds of 5 selected accessions (Tables 1, 2; Figs. 1, 2 and 3). The seed oil content (50.76–60.88%) of *P. chinensis* from all accessions (Fig. 2a) was not only greater than that of other traditional woody oil plants (11.68–40.28%) [12–14, 37–44], but also higher than that reported for *P. chinensis* seeds from other germplasms (42.5–50.0%) [17–19], implying that *P. chinensis* accessions, especially for high oil-bearing PC-HN (60.88%) and PC-SX (55.37%), had great potential as biodiesel feedstock. This, integrated with high yield of biodiesel from seed oils of PC-HN (98.15%) and PC-SX (96.76%) (Table 2) could satisfy the standard of EN 14,214 (96.5%), indicated that the *P. chinensis* seed oils of PC-HN and PC-SX accessions may be advantage for high-yield biodiesel production. It is worth noticing that the high-quality oils for ideal biodiesel production should contain suitable amount of SFA, high percentage of MUFA and low level of PUFA [12, 16, 31–33, 45]. In this work, all accession seeds were detected with high content of MUFA (42.80–70.72%) and low levels of PUFA (18.78–43.35%) and SFA (10.50–14.54%) (Table 1), and importantly, the total amount of C18:1 and C18:2 in all accessions accounted for more than 81% of seed oils (Table 1), emphasizing that the seed oils from all accession may be as raw material suitable for biodiesel, corresponded to previous results of several traditional woody oil plants [12, 46–50]. Considering that accession PC-HN had the maximum content of MUFA (70.72%), the minimum amounts of PUFA (18.78%) and SFA (10.50%) and a small ratio (0.252) of C18:2/C18:1 (Fig. 3a, b), together with superior value of IV (98.15), CN (51.87), OS (3.57 h), CP (-6.28 °C) and CFPP (-8.90 °C) in biodiesel (Table 2), it seems certain that the seed oils from PC-NH with superior FA compositions could satisfy the requirement for high-quantity biodiesel production.

### LEC1/WRI1-mediated transcription regulation for high oil production in seeds of* P. chinensis* and transgenic lines

In oil plants, FA synthesis and oil accumulation as a part of seed development, involving a series of gene expression, is highly regulated by several TFs [22–29, 51–59]. In this work, of our recent annotated 12 TFs (LEC1, ABI3, FUS3, WRI1, VAL1, L1L, GL2, AP2, VAL2, VAL3, EMF2 and DOF1) in *P. chinensis* seeds by transcriptome analysis [17], only both LEC1 and WRI1 transcripts increased and coincided with seed oil content across all accessions (Fig. 4a). Given that LEC1 and its targeted WRI1 at the center of interaction network were highly correlated with oil-synthetized enzymes (Fig. 4b), it seems clear that both WRI1 and LEC1 may act as critical positive regulators crucial for gene transcriptional expression relevant to seed oil accumulation of *P. chinensis* across all accessions, as was noted for several oil plants [29, 60–63]. Of note, WRI1, has been shown to control the expression of targeted genes involved in glycolysis, FA synthesis and TAG accumulation during oilseed development [29, 53, 57, 64–66]. This was clearly demonstrated by our findings that the abundantly coordinated transcripts of the enzymes relevant for carbon allocation (plastidic glycolysis and acetyl-CoA formation), FA synthesis, TAG assembly and oil storage in developing seeds of transgenic *PcWRI1* lines (Fig. 10) were temporally associated with the increased amount of seed oils (Fig. 7b), emphasizing that these genes, as the targets of WRI1, were sequentially and transcriptionally activated by *PcWRI1* overexpression, giving rise to a strong collaborative funneling of carbon and acyl flux into oil biosynthetic pathway for high oil production in developing seeds of transgenic *PcWRI1* lines, destined for an 23.59% increase in oil content of ripen seed as compared with the WT (Fig. 7b). This was consistent with former results of other oilseed plants [52, 53, 65, 67–71], and also evidenced by the fact of an increase in FA synthesis and oil accumulation by ectopic overexpression of *WRI1* from several oilseed plants (such as rapeseed, oil palm, sunflower, coconut, maize, soybean, *Camelina sativa* and *J*. *curcas*) [22–24, 27, 28, 65–67, 70, 72].

As a crucial TF governing plant oil accumulation, WRI1 is known to be up-regulated by LEC1/2, ABI3 or FUS3 [67]. Here, a similar pattern of high transcript for LEC1 and WRI1 in *P. chinensis* seeds across all accessions (Fig. 4a) revealed *WRI1* as one target gene of LEC1, which was also identified in *A. thaliana* and *P. sibirica* seeds [14, 51], but contrasted with the idea that WRI1 function may be independent of upstream TFs in controlling oil biosynthesis in oil palm [68]. LEC1 has been shown as a central regulator in TF-mediated regulation network for oil biosynthesis by transcriptional control distinct gene sets during seed development [62, 64, 73, 74], and *LEC1* overexpression increased oil content by up-regulating several genes relevant for FA and oil synthesis in oil plants [25, 54, 64, 74, 75], but *lec1* loss-of-function mutation represented suppression of oil accumulation [64]. In this study, the transcripts of endogenous oil-related TFs (AtABI3, AtWRI1, AtFUS3 and AtL1L) were significantly up-regulated in developing seeds of transgenic *PcLEC1* lines (Fig. 9c-f), showing the same temporal co-transcription of oil biosynthetic genes (Fig. 10c-e), reflecting indeed that ectopic expression of *PcLEC1* could promote transcript of its targets (*AtABI3*, *AtWRI1*, *AtFUS3* and *AtL1L*). Given that a higher root length, plant height, and rosette and leaf size was detected in transgenic *PcLEC1* line than in transgenic *PcWRI1* line (Fig. 6), integrated with the relatively low amount of seed oil (Fig. 7b) and low transcript level of oil accumulation-related enzymes (carbon flux allocation, FA synthesis, TAG assembly and oil storage) in developing seeds of transgenic *PcLEC1* line compared with the transgenic *PcWRI1* lines (Fig. 10), it seems certain that overexpression of *PcLEC1* may contribute mostly to plant development of transgenic line, but an increase of seed oil content may be likely attributed to *PcWRI1* overexpression.

Overall, the strong correlation of seed oil accumulation (Fig. 7b) with rich transcript of the enzymes essential for oil biosynthetic process (carbon allocation, FA synthesis, TAG assembly and oil body formation) in developing seeds of transgenic lines with *PcLEC1* or *PcWRI1* (Fig. 10) revealed that LEC1/WRI1-mediated transcription regulatory network may play a major role in plant growth, seed development and oil accumulation.

## Conclusions

In this work, the cross-accessions comparisons on seed weight, oil content, FA composition, biodiesel yield, and fuel properties were conducted to determine ideal accession PC-HN as novel promising feedstock for superior biodiesel production. The application of an integrated assay on the cross-accessions correlation of seed oil accumulative amount with TF transcript level, and protein interaction between the TFs and oil-synthesis enzymes, has led to identification of two key TFs (LEC1 and WRI1) involved in LEC1/WRI1-mediated regulatory network essential for high-quality seed oil accumulation of *P. chinensis* of different accessions. Notably, the transgenic results indicated that overexpression of *PcWRI1* or *PcLEC1* from *P. chinensis* seeds in Arabidopsis could facilitate seedling growth and seed development, and increase transcripts of several genes relevant for carbon allocation (plastidic glycolysis and PDC), FA synthesis (ACC, BCCP1/2, MAT, FATA/B, KAS I/II/III, EAR and SAD6), TAG assembly (LACS8/9, GPAT9, LPAAT4, PAP2 and DGAT1) and oil body formation (OLEO1/2/3/5), leading to an increase in ripen seed oil content (17.73%-23.59%) and MUFA amount (42.44%-50.73%), destined for biodiesel property improvement. Our findings may present strategies for developing *P. chinensis* seed oils as biodiesel feedstock, and highlight translational potential of PcWRI1 or PcLEC1 for further bioengineering of high oil yield and quality destined for ideal biodiesel in *P. chinensis* and other oilseed plants.

## Materials and methods

### Collection of fruit samples of *P. chinensis* and detections of seed size and weight

Based on our previous studies on 18 provenances of *P. chinensis* located in south of Qinling Mountains (geographical coordinates approximately E113°00′-119°26′, N28°26′-33°49′) [17, 19], a total of 5 plus trees (defined as superior trees) of *P. chinensis* (germplasm accessions PC-BJ, PC-AH, PC-SX, PC-HN and PC-HB) with high seed yield were selected and identified, and deposited in the Forest and Flower Germplasm Resource Genebank of Beijing Forestry University in China (Voucher No. 1111C0003102003374, 1111C0003102003402, 1111C0003102003408, 1111C0003102003113 and 1111C0003102003108, respectively). The ripen fresh fruits were collected from 10-year-old tree, and 4000 fruits from 10 trees (400 fruits each tree) of each accession were randomly selected each time. After removing the sarcocarp, the undamaged and plump fresh seeds were selected and immediately frozen in liquid nitrogen and stored at -80 °C until use for FA detection, qRT-PCR assay and gene cloning. The size and weight of seeds were respectively detected with vernier caliper and electronic balance. All analyses were conducted in triplicate.

### Plant material and growth conditions

The wild type (WT) Arabidopsis was Col-0 ecotypes, and the seeds of WT and transgenic line with the empty vector pCAMBIA2300*/CaMV35S*::*GUS* (null transgenic, NT) were provided by our laboratory. To prepare Arabidopsis plants for genetic transformation of *PcWRI1* and *PcLEC1*, the WT seeds were surface-sterilized and germinated on half-strength MS medium, and imbibed in the dark for 3–4 d at 4 °C, and then were placed into an incubator under a continuous artificial light period of 16 h (22 °C) at a photon flux density of 200 μmol m^−2^ s^−1^ and a dark period of 8 h (18 °C). After 20 d, the seedlings were transplanted into the pots containing a soil mixture (humus-soil/vermiculite/perlite, 3:3:1, *v*/*v*/*v*) and grown under a condition of 16 h photoperiod (24 °C) and 8 h dark period (22 °C) for gene transformation and seed harvest.

### Section and observation of seed lipid droplet of *P. chinensis*

The mature seeds of each accession (PC-BJ, PC-AH, PC-SX, PC-HN and PC-HB) used for lipid droplet observation were horizontally and vertically transected, and colored by safranin with fast green. The resulting sections were scanned by PANNORAMIC MIDI (3DHISTECH, Hungary), and the density of lipid droplet was calculated by Image J software 1.8.0. Ten individual seeds were measured in each accession.

### Oil extraction and trans-esterification

The analyses of seed oil content and FA compositions were conducted on the *P*. *chinensis* seeds of all accessions, and the developing seeds of WT, NT, and T3 generation of *PcWRI1* and *PcLEC1* transgenic Arabidopsis lines.

About 10 g of the undamaged and plump dry seeds of *P*. *chinensis* (3 samples per accession) were pulverized into powders with domestic grinder and then lyophilized by freeze dryer (LGJ-12, China) to extract oil with petroleum ether using Soxhlet apparatus at 45–50 °C for 6–8 h [16], and then the oils were separated from organic mixture by rotary evaporator and dried to constant in ventilated oven at 105 °C. The content of extracted oil from each accession seed was calculated as the difference between the weights of seed sample before and after extraction, and expressed as the percentage of the extracted oil weight to dry seed weight (%, g/g). To analyze FA compositions, the seed oils from each accession were *trans*-esterified as previously described [16].

A total of 20 Arabidopsis seeds (per developing period) were weighted and trans-methylated at 95 °C for 1.5 h in 1 mL of methanol (containing 50 μL H_2_SO_4_, 50 μg BHT and 25 0 μg C17:0) and 300 μL toluene, and then 1.5 mL NaCl solution (0.9%, *m*/*v*) was added. The obtained oils were recovered by three sequential extractions with 1 mL of hexane, and evaporated under a stream of nitrogen and re-dissolved in 50 μL hexane. The oil contents were calculated by the following equations [76]: Oil content (%, g/g) = 100 × [Σ*A*_*i*_ + 4 × (Σ*A*_*i*_ /*MW*_*i*_)/3]/*W*. Where* A*_*i*_ is the weight of the *i*th FAME, *MW*_*i*_ is the molecular mass of each component, *W* is the seed weight of *A*. *thaliana*, 4 is the *M*_*r*_ difference between three moles of FAME and TAG.

All the determination was conducted for three biological replicates of seed sample with three technical replicates.

### Analyses of FAMEs and biodiesel yield of* P*.* chinensis* seed oils

The FA methyl esters (FAMEs) obtained from each accession seed was used to detect FA compositions by Agilent 6890 (California, USA) gas chromatograph equipped with flame ionization detector (GC-FID) [16]. The HP-INNOWax capillary column (inner diameter 0.32 mm, filmthickness 0.5 μm, split 1:20) was used, and the temperature was programmed at 60 °C, with a rise of 4 °C min^−1^ to 220 °C and heated to 240 °C for 10 min. The carrier gas was helium with a flow rate of 1.0 mL min^−1^. The peaks of FAMEs were identified by comparing their retention time with that of the known standards, and peak integration was performed by applying HP3398A software. Each FAME assay was performed in triplicate. The biodiesel yield from *P*. *chinensis* seed oils was calculated by the previous method [12, 16], where the yield was defined as the percentage (%, g/g) of the obtained total amount of FAMEs (g) to the used amount of raw oils (g).

### Evaluation of biodiesel fuel property of* P*.* chinensis* seed oils

The biodiesel fuel properties (CN, IV, CFPP, CP, OS, KV and D) of *P*. *chinensis* seed oils from 5 accessions were evaluated by the FAME compositions according to our previously method [31]. The biodiesel fuel characteristics of raw oils were compared with the relevant biodiesel standards of EN 14,214–2008 (European, 2008), ASTM D6751-2010 (USA, 2010), DIN V51,606-1997 (Germany, 1997) and GB/T 20,828–2007 (China, 2007).

### Protein interaction network analysis

Based on previous transcriptome data of *P. chinensis* seeds [17], the Retrieval of Interacting Genes/Proteins (STRING version 9.0, http://string90.embl.de/) was employed to analyze the potential interactions between all our identified TFs and functional proteins involved in FA synthesis and oil accumulation. STRING analysis was conducted using high confidence (score 0.7), and cluster analysis was performed by using *k*-means with a value of *k* = 3.

### Isolation and structural analysis of *PcWRI1* and *PcLEC1*

Total RNA isolated from *P. chinensis* seeds by RNeasy Plant Mini Kit (Qiagen, Inc., USA) was used to synthesize cDNA by PrimeScript™ RT reagent Kit (TaKaRa Dalian, China). The full-length sequences of *PcWRI1* and *PcLEC1* were amplified using the primers of PcWRI1-U/PcWRI1-D and PcLEC1-U/PcLEC1-D, respectively (Additional file 11: Table S9), and then subcloned into pGEM-T vector (Promega, USA) for sequencing. Multiple sequence alignment and phylogenetic tree were constructed by DNAMAN 7.0 with ClustalX program and RAxML-NG (https://raxmlng.vital-it.ch/) with maximum likelihood method, respectively [77]. The 3D model for PcWRI1 and PcLEC1 as well as homologous proteins from *A*. *thaliana*, *Z*. *may* and *G*. *max* was constructed by online tool Phyre2 (http://www.sbg.bio.ic.ac.uk/phyre2/html/).

### Heterologous expression of* PcWRI1* and *PcLEC1 in Arabidopsis thaliana*

The ORFs of *PcWRI1* and *PcLEC1* were amplified with the primer pairs PcWRI1-F-*EcoR*I/PcWRI1-R-*BamH*I and PcLEC1-F-*Kpn*I/PcLEC1-R-*Xba*I, and subcloned into pCAMBIA2300 driven by the *CaMV35S* promoter to construct the vectors of pCAMBIA2300/*CaMV35S*::*PcWRI1* and pCAMBIA2300/*CaMV35S*::*PcLEC1*, and then transformed into *Agrobacterium tumefaciens* strain EHA105 by freeze–thaw method and delivered into wild-type Arabidopsis plants (ecotype Col-0) by floral dip method [78]. The transgenic lines were screened on 1/2MS medium (containing 50 μg/mL kanamycin, 20 g/ L sucrose and 20 g/L agar) and detected by PCR to obtain T3 seeds. Finally, 3 independent-homogenous transgenic lines of *PcWRI1* and *PcLEC1* were obtained and examined by χ^2^ test for further analyses.

### Analyses of plant phenotypic and seed traits

To explore the effect of *PcWRI1* or *PcLEC1* overexpression on phenotypes of transgenic lines, a comparative analysis of morphological features (root length, plant height, leaf size and seed weight) was conducted on the seedlings of WT, NT and T3 transgenic Arabidopsis lines (*35S*::*PcWRI1* and *35S*::*PcLEC1*). The root length of 10-day-old seedling germinated on half-strength MS medium was detected. The 20-day-old seedlings were transfer to pots and grown in separate flats in same incubator used for detections of plant height and flowering time. One thousand dry seeds from the controls (WT and NT) and T3 transgenic lines were weighed, and the results was expressed as thousand seed weight (mg/1000 seeds). All the determination was conducted in triplicate.

To harvest Arabidopsis seeds at different developing stages, the flowers on main axis were tagged on the day of anthesis, and then the seeds (10 plants of WT and transgenic lines per set) were collected at 5 developing stages from 4 to 20 DAA with an interval of 4 days. The flowers, leaves, stems and roots were collected at 0 DAA. All samples were frozen in liquid nitrogen and stored at − 80 °C for analyses of oil content, FA compositions, RNA extraction and qRT-PCR.

### Construction of prediction model for biodiesel property of raw oils from ripen seeds of transgenic Arabidopsis

Triangular prediction model for biodiesel property of raw oils from ripen seeds of transgenic lines was constructed based on the influence of FA compositions [12, 16]. To predict biodiesel properties of seed oils from transgenic lines, the percentages of SFA, MUFA and PUFA in ripen seed oils from each transgenic line were calculated to outline triangular prediction graph, in which three angular points of the triangle meant the 100% of SFA, MUFA and PUFA, respectively. In triangular graph, the region existed at the far end of the polyunsaturated angular point (lower left vertex) and the saturated angular point (lower right vertex) was delineated to predict the biodiesel fuel properties, taking into account the CN, IV, CFPP and OS [12, 16].

### Gene expression analysis by qRT-PCR

Total RNA was extracted from *P*. *chinensis* seeds by RNeasy Plant Mini Kits (Qiagen) and quantified by Nanodrop ND-1000 spectrophotometer (N Wilmington, USA) to be reverse-transcribed by Reverse Transcription System (Promega). qRT-PCR was conducted on 7500 Real-Time PCR System using SYBR Premix Ex Taq Kit (TaKaRa). All amplified primers (Additional file 12: Table S10) were designed by PrimerQuest (http://www.idtdna.com/PrimerQuest/Home/Index). The genes encoding large subunit ribosomal protein L32e (RPL32e) and ubiquitin-conjugating enzyme (UBC) were used as inner references [16, 79] for the detection of TF gene transcription in *P*. *chinensis* seeds, and its expression level in the seeds of different accessions was set to 1.00 for standardization. As for detections of gene expression in developing seeds of the controls (WT and NT) and transgenic lines, Arabidopsis *ACTIN* gene was used as endogenous reference, and expression level from seed sample at 4 DAA was set to 1.00 for standardization. Three biological replicates with three technical repetitions each were performed for all qRT-PCR assays.

## Supplementary Information

**Additional file 1.**

**Additional file 2.**

**Additional file 3.**

**Additional file 4.**

**Additional file 5.**

**Additional file 6.**

**Additional file 7.**

**Additional file 8.**

**Additional file 9.**

**Additional file 10.**

**Additional file 11.**

**Additional file 12.**

## Data Availability

All data generated or analyzed during this study are included in this published article and its Additional files.

## Abbreviations

ABI3: ABSCISIC ACID INSENSITIVE3
ACC: Acetyl-CoA carboxylase
CFPP: Cold filter plugging point
CN: Cetane number
CP: Cloud point
DGAT1: Acyl-CoA:DAG acyltransferase 1
EAR: Enoyl-ACP reductase
FAD: Fatty acid (FA) desaturase
FATA/B: Fatty acyl-ACP thioesterase A/B
FAMEs: FA methyl esters
FUS3: FUSCA3
GPAT: Acyl-CoA:G3P acyltransferase
KAS I/II/III: 3-Ketoacyl ACP synthase I/II/III
L1L: LEAFY COTYLEDON1-LIKE
LACS: Long-chain acyl CoA synthetase
LEC1: LEAFY COTYLEDON1
LPAAT2: Acyl-CoA:LPA acyltransferase 2
MAT: Malonyl-CoA-ACP transferase
OLEO: Oleosin
PDAT: Diacylglycerol (DAG) acyltransferase
PAP: PA phosphatase
PDC: Pyruvate (PYR) dehydrogenase complex
PFK: Phosphofructokinase
PK: PYR kinase
qRT-PCR: Quantitative real time-PCR
SAD6: 18:0-ACP desaturase 6
TAG: Triacylglycerol
WRI1: WRINKLED1
