# Supplementary material for: Determination of superior Pistacia chinensis accession with high-quality seed oil and biodiesel production and revelation of LEC1/WRI1-mediated high oil accumulative mechanism for better developing woody biodiesel
Source: BMC Plant Biol. 2023 May 19;23:268. doi: 10.1186/s12870-023-04267-y (PMC10197815; doi:10.1186/s12870-023-04267-y)
Supplement: Supplementary file 4 — Additional file 4. [file 12870_2023_4267_MOESM4_ESM.docx]

**

 Figure S1. The original** **full-length gel images matched to the cropped versions in Figure 5 of the manuscript. (a)** The **u**nprocessed gel image matched with the cropped version in Fig. 5a. **(b)** The unprocessed gel image matched with the cropped version in Fig. 5b. **(c)** The unprocessed gel image matched with cropped version in Fig. 5d. **(d)** The unprocessed gel image matched with cropped version in Fig. 5e. **(e)** The unprocessed gel image matched with the cropped version in Fig. 5h. **(f)** The unprocessed gel image matched with the cropped version in Fig. 5i.
