# Supplementary material for: Determination of superior Pistacia chinensis accession with high-quality seed oil and biodiesel production and revelation of LEC1/WRI1-mediated high oil accumulative mechanism for better developing woody biodiesel
Source: BMC Plant Biol. 2023 May 19;23:268. doi: 10.1186/s12870-023-04267-y (PMC10197815; doi:10.1186/s12870-023-04267-y)
Supplement: Supplementary file 5 — Additional file 5. [file 12870_2023_4267_MOESM5_ESM.docx]

**
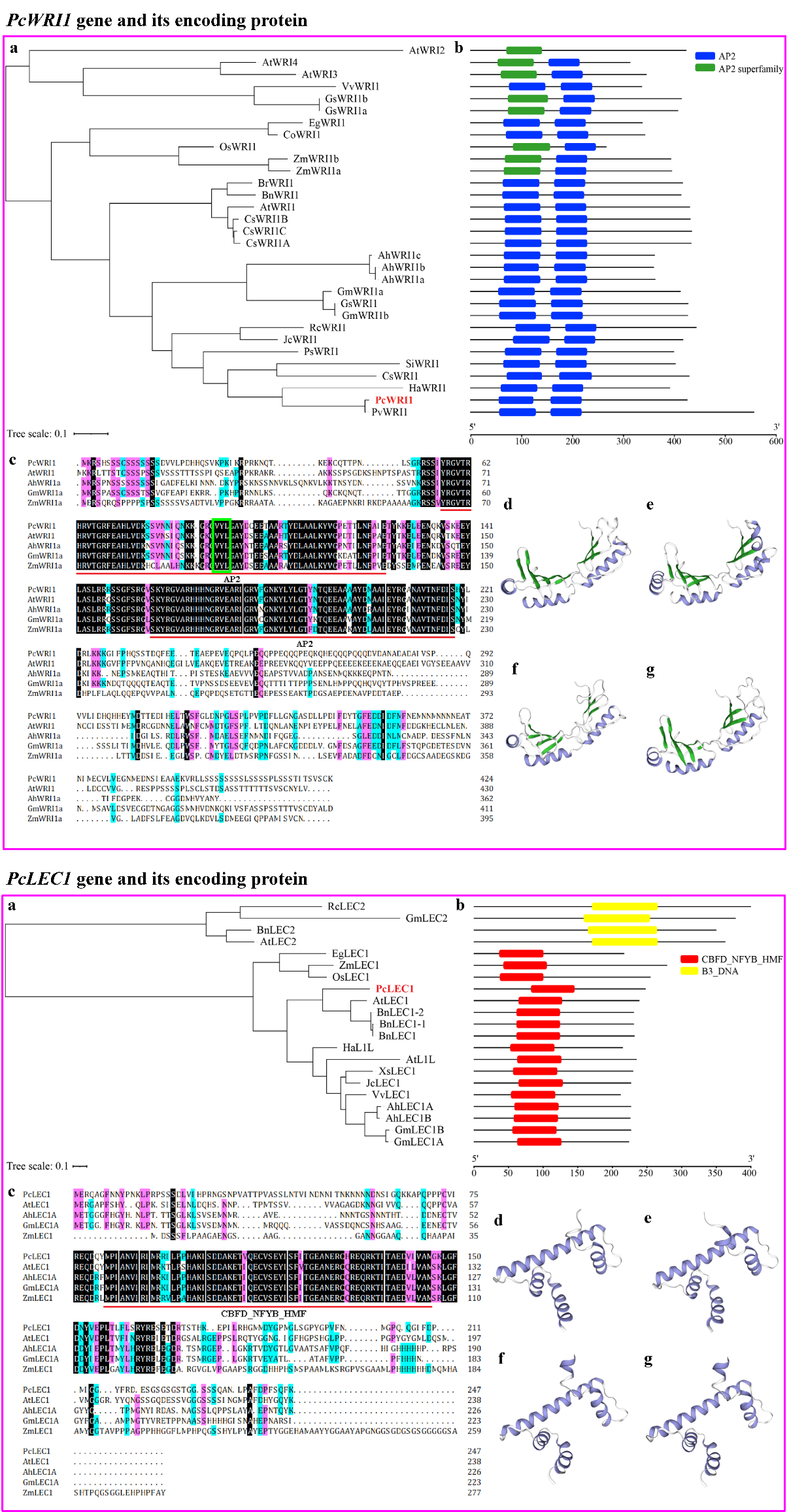
**

**Figure S2.** **Bioinformatics analyses of PcWRI1 and PcLEC1 protein from *Pistacia chinensis* seeds.** **(a)** Phylogenetic analysis of PcWRI1 with 30 other WRI1 or WRI1-like proteins. Accession numbers for WRI1 or WRI1-like proteins used here were listed: *Arachis* *hypogaea* (AhWRI1a, XP_025673174.1; AhWRI1b, XP_025673175.1; AhWRI1c, XP_025615545.1), *Arabidopsis thaliana* (AtWRI1, NP_001030857.1; AtWRI2, ABG25074.1; AtWRI3, NP_563990.1; AtWRI4, NP_001077849.1), *Brassica* *napus* (BnWRI1, ABD16282.1), *Cocos* *nucifera* (CoWRI1, AFH68065.1), *Brassica* *rapa* (BrWRI1, XP_009103913.1), *Camellia* *sinensis* (CsWRI1, XP_028103108.1; CsWRI1A, AQP31129.1; CsWRI1B, AQP31130.1; CsWRI1C, AQP31131.1), *Elaeis* *guineensis* (EgWRI1, AHX71677.1), *Glycine* *soja* (GsWRI1, RZB98349.1), *Glycine* *max* (GmWRI1a, NP_001236002.2; GmWRI1b,NP_001357993.1), *Jatropha* *curcas* (JcWRI1, NP_001292947.1), *Helianthus* *annuus* (HaWRI1, XP_022005216.1), *Oryza* *sativa* (OsWRI1 ,CAE00853.1), *Prunus* *sibirica* (PsWRI1, AIW62177.1), *Pistacia* *vera* (PvWRI1, XP_031260581.1), *Ricinus* *communis* (RcWRI1, BAM75178.1), *Sesamum* *indicum* (SiWRI1, XP_011078716.1), and *Zea* *mays* (ZmWRI1a, ACF83189.1; ZmWRI1b, ACF80269.1). **(b)** Domain assay for the corresponding WRI proteins. The AP2 and AP2 superfamily domain were shown as blue and green boxes, respectively. Numbers indicate the amino acid positions along the proteins. **(c)** Multiple alignment for amino acid sequences of WRI1 proteins from *P*. *chinensis*, *A*. *thaliana*, *A*. *hypogaea*, *G*. *max* and *Z*. *mays*. The underlined regions were the DNA binding AP2 domains, the green box was the key ‘VYL’ motif, and the letters with black and other colors showed identical and similar amino acids, respectively. **(d)** Prediction of 3D model for the conserved domains of PcWRI1. **(e)** Prediction of 3D model for the conserved domains of AtWRI1. **(f)** Prediction of 3D model for the conserved domains of GmWRI1a. **(g)** Prediction of 3D model for the conserved domains of ZmWRI1a. **(h)** Phylogenetic assay of PcLEC1 with 16 LEC1 homologous proteins and 4 LEC2 proteins from *A*. *thaliana*, *B*. *napus*, *G*. *max* and *R*. *communis*. Accession numbers were listed: *A*. *hypogaea* (AhLEC1A, AJP62202.1; AhLEC1B, AJP62203.1), *A*. *thaliana* (AtL1L, AAN15924.1; AtLEC1, Q9SFD8.2; AtLEC2, AAL12005.1), *B*. *napus* (BnLEC1, ACB12186.1; BnLEC1-1, ADF81044.1; BnLEC1-2, ADF81045.1; BnLEC2, ADO16343.1), *E*. *guineensis* (EgLEC1, BBH96243.1), *H*. *annuus* (HaL1L,XP_022018583.1), *G*. *max* (GmLEC1A, NP_001236625.1; GmLEC1B, NP_001239679.1; GmLEC2, ATL76750.1), *J*. *curcas* (JcLEC1, NP_001295673.1), *O*. *sativa* (OsLEC1, AAP22065.1), *Pistacia chinensis* (PcLEC1, ADK91820.1), *R*. *communis* (RcLEC2, AGT56425.1), *Vitis vinifera* (VvLEC1, AUJ18469.1), *Xanthoceras sorbifolium* (XsLEC1, AYD60585.1), and *Z*. *mays* (ZmLEC1, AAK95562.1). **(i)** Motif assay for corresponding LEC1 and LEC2 proteins. The CBFD_NFYB_HMF and B3 motifs were shown as red and yellow boxes, respectively, and the numbers indicate amino acid positions along the protein. **(j)** Multiple alignment for amino acid sequences of LEC proteins from *P*. *chinensis*, *A*. *thaliana*, *A*. *hypogaea*, *G*. *max* and *Z*. *mays*. The underlined region was the CBFD_NFYN_HMF domain, the letters with black and other colors showed identical and similar amino acids, respectively. (k) Prediction 3D model for PcLEC1. **(l)** Prediction 3D model for AtLEC1. **(m)** Prediction of 3D model for GmLEC1A. **(n)** Prediction of 3D model for ZmLEC1.
