# Supplementary material for: Determination of superior Pistacia chinensis accession with high-quality seed oil and biodiesel production and revelation of LEC1/WRI1-mediated high oil accumulative mechanism for better developing woody biodiesel
Source: BMC Plant Biol. 2023 May 19;23:268. doi: 10.1186/s12870-023-04267-y (PMC10197815; doi:10.1186/s12870-023-04267-y)
Supplement: Supplementary file 6 — Additional file 6. [file 12870_2023_4267_MOESM6_ESM.docx]

**Table S4** **Statistical result of chi-square test for the screening of T3 homozygotes of transgenic Arabidopsis lines with overexpression of *PcWRI1* or *PcLEC1***

| Line | Total | Number of plants | | Ratio | *χ*^2^ | *P* value | Result |
| --- | --- | --- | --- | --- | --- | --- | --- |
|  |  | Resistant plant | Sensitive plant |  |  |  |  |
| PcWRI1-1 | 360 | 360 | 0 | 1:0 | 0 | >0.99 | Homozygote |
| PcWRI1-2 | 358 | 356 | 2 | 1:0 | 0.01 | 0.95-0.90 | Homozygote |
| PcWRI1-3 | 361 | 360 | 1 | 1:0 | 0.00 | 0.98-0.95 | Homozygote |
| PcWRI1-4 | 361 | 260 | 101 | 3:1 | 1.71 | 0.20-0.10 | Heterozygote |
| PcWRI1-5 | 359 | 276 | 83 | 3:1 | 0.68 | 0.50-0.30 | Heterozygote |
| PcLEC1-1 | 357 | 357 | 0 | 1:0 | 0.00 | >0.99 | Homozygote |
| PcLEC1-2 | 361 | 361 | 0 | 1:0 | 0.00 | >0.99 | Homozygote |
| PcLEC1-3 | 359 | 358 | 1 | 1:0 | 0.00 | 0.98-0.95 | Homozygote |
| PcLEC1-4 | 361 | 283 | 78 | 3:1 | 2.22 | 0.20-0.10 | Heterozygote |
| PcLEC1-5 | 360 | 257 | 103 | 3:1 | 2.50 | 0.20-0.10 | Heterozygote |
